# Alkaline magmas in shallow arc plutonic roots: a field and experimental investigation of hydrous cumulate melting in the southern Adamello batholith

**DOI:** 10.1007/s00410-023-02047-3

**Published:** 2023-09-03

**Authors:** Manuel Pimenta Silva, Felix Marxer, Tobias Keller, Andrea Giuliani, Peter Ulmer, Othmar Müntener

**Affiliations:** 1 Institute of Geochemistry and Petrology, ETH Zürich, Clausiustrasse 25, 8092 Zurich, Switzerland; 2 Institute of Mineralogy, Leibniz University Hannover, Callinstraße 3, 30167 Hannover, Germany; 3 Institute of Earth Sciences, Université de Lausanne, Lausanne, Switzerland

**Keywords:** Arc magmatism, Chemical diversity, Cumulate melting, Thermal modelling, Experimental petrology, Nepheline-normative rocks, Petrological cannibalism, Arc batholiths, Magma plumbing systems, Amphibole

## Abstract

Despite the first-order importance of crystallisation–differentiation for arc magma evolution, several other processes contribute to their compositional diversity. Among them is the remelting of partly crystallised magmas, also known as cumulate melting or ‘petrological cannibalism’. The impact of this process on the plutonic record is poorly constrained. We investigate a nepheline-normative dyke suite close to the Blumone gabbros, a large amphibole-gabbro unit of the Tertiary Southern Alpine Adamello igneous complex. The compositions of the studied dykes are characterised by low SiO_2_ (43–46 wt. %), MgO (5.0–7.2 wt. %), Ni (18–40 μg/g), and high Al_2_O_3_ (20.2–22.0 wt. %) contents. Phenocrystic plagioclase in these dykes exhibits major, trace, and Sr isotope compositions similar to Blumone cumulate plagioclase, suggesting a genetic link between the nepheline-normative dykes and the amphibole-gabbro cumulates. We tested this hypothesis by performing saturation experiments on a nepheline-normative dyke composition in an externally heated pressure vessel at 200 MPa between 975 and 1100 °C at fO_2_ conditions close to the Ni–NiO buffer. Plagioclase and spinel are near-liquidus phases at and above 1050 °C, contrasting with the typical near-liquidus olivine ± spinel assemblage in hydrous calc-alkaline basalts. The alkaline nature of the dykes results from the abundance of amphibole in the protolith, consistent with melting of amphibole-gabbro cumulates. We modelled the heat budget from the repeated injection of basaltic andesite into a partly crystallised amphibole-gabbro cumulate. The results of this model show that no more than 7% of the cumulate pile reaches temperatures high enough to produce nepheline-normative melts. We propose that such nepheline-normative dykes are a hallmark of hydrous cumulate melting in subvolcanic plumbing systems. Therefore, ne-normative dykes in arc batholiths may indicate periods with high magma fluxes.

**Supplementary Information:**

The online version contains supplementary material available at 10.1007/s00410-023-02047-3.

## Introduction

Many studies support the incremental construction of crustal plutonic magma reservoirs by recurrent melt inputs and thermal reactivation over timescales ranging from tens of thousands to millions of years (e.g., Coleman et al. 2004; Schoene et al. 2012; Schaltegger et al. 2019). Besides the consequences of such reactivation for physical properties (e.g., magma density and viscosity), the intrusion of new magma batches with potentially different composition and volatile content will increase the already substantial chemical diversity of arc magmas. Evidence of reactivation processes includes dissolution–precipitation of solid phases upon entrainment into melt (e.g., Tsuchiyama 1985; Blundy and Shimizu 1991; Cashman and Blundy 2013), melt hybridisation (Eichelberger 1975; Pyle et al. 1988; Reubi and Blundy 2009), and partial melting of cumulate fragments (plutonic recycling; e.g., Dungan and Davidson 2004; Ratajeski et al. 2005; Reubi and Blundy 2008).

The latter has been addressed by analysis of melt inclusions (e.g., Schiano et al. 2000; Reubi and Blundy 2008) or interstitial melt (e.g., Costa et al. 2002; Dungan and Davidson 2004) in volcanic products. Despite the apparently small volumes of cumulate remelting products, evidence for this process implies that the evolving magmatic system experienced thermal and/or chemical perturbation (e.g., Pistone et al. 2016, 2017; Neave et al. 2021). Recycling of mafic roots has been investigated at the Volcán Colima (Reubi and Blundy 2008), Tatara-San Pedro complex (Dungan and Davidson 2004), Volcán Llaima (Reubi et al. 2011), or Batan island (e.g., Schiano et al. 2000). Dungan and Davidson (2004) identified this process by observing LILE enrichment in the lavas that is inconsistent with fractional crystallisation but agrees with phlogopite–amphibole–plagioclase melting. Reubi et al. (2011) identified plutonic root assimilation in the Volcán Llaima (Chile) based on U-Series disequilibria in a setting where other evidence in major, trace, or isotopic proxies is lacking. Moreover, the generation of ultra-calcic, nepheline-normative melts, found in intra-oceanic arc settings, has been attributed to the partial melting of pargasite wehrlites (Médard et al. 2006; Sorbadère et al. 2013). Studying the plutonic record allows quantification of the proportion of products formed by cumulate melting relative to fractional crystallisation and/or mixing and hybridisation with mafic input. To our knowledge, studies of cumulate melting in the plutonic arc record are limited to the El Capitan complex (central Sierra Nevada, USA), where Ratajeski et al. (2001, 2005) identify products of low-temperature gabbroic cumulate melting in the deep crust, which deviate from a typical arc liquid line of descent. In all these examples, partial melting of hydrous phases, mostly amphibole and/or biotite, is fundamental to this process.

The Blumone complex in the tertiary southern Adamello batholith (Southern Alps; Northern Italy) has been recognised as a subvolcanic plumbing system where ascending magmas interact with a cogenetic mafic cumulate complex (Ulmer et al. 1983; John and Blundy 1993; Schoene et al. 2012). Consequently, several syn-plutonic features typical of incremental intrusions are observed, such as the occurrence of ultramafic xenoliths from deeper crustal levels (Ulmer et al. 1983) or mingling between different magma batches (e.g., Callegari and Dal Piaz 1973; Callegari and Brack 2002). The interaction of newly intruded magma with previously formed cumulates is observed at the crystal scale, where high-An plagioclase cores exhibit resorption textures (Blundy and Shimizu 1991). The diversity of reworking features and the 2 km vertical relief make the 42 Ma-old Blumone complex ideal for studying cumulate melting in a plutonic setting.

In this study, we characterise a suite of dykes with nepheline-normative compositions (i.e., SiO_2_-undersaturated), which show contrasting geochemical properties to the well-documented differentiation suite in the Adamello batholith. These dykes outcrop in the vicinity of an amphibole gabbroic cumulate complex. Since amphibole has a low SiO_2_ content, we hypothesise that the petrogenesis of the Blumone nepheline-normative dykes involved the melting of amphibole-rich cumulates. To address this hypothesis, we perform a detailed petrological study of these dykes. We measure the major, trace, and isotopic composition of minerals in the nepheline-normative dykes and in the amphibole cumulates to assess their hypothesised genetic link. By performing isobaric saturation experiments at 200 MPa, we determine the liquidus temperature of the anomalous dyke compositions and their near-liquidus mineral phase relationships. Based on the experimental results, we developed a 1D thermal model to constrain the potential extent of high-temperature cumulate melting in subvolcanic conduits.

Our results support the hypothesis of generating the nepheline-normative dykes by high-temperature melting of amphibole gabbroic cumulates. We discuss the importance of nepheline-normative magmas and the dependence of their genesis on magmatic flux rates. Although cumulate melting is not a primary differentiation mechanism, it contributes to the chemical diversity observed in arc magmas. Identifying and understanding such phenomena are crucial, particularly when the geometry of the magmatic system cannot be fully constrained, as is, for example, the case with the melt inclusion record.

## Geological setting

The Adamello Massif (Northern Italy) is an Alpine calc-alkaline batholith resulting from the early- to mid-Tertiary subduction of the European plate below the Adriatic microplate (Callegari and Brack 2002). The batholith is predominantly composed of intermediate granitoids accompanied by minor mafic intrusions, which intruded into the Southern Alpine basement and the overlying Permo-Mesozoic sedimentary units at a pressure of ca. 0.2–0.3 GPa (e.g., Nimis and Ulmer 1998; Callegari and Brack 2002). It was incrementally built from ca. 42 to ca. 31 Ma and shows a progressive SW–NE decrease in age (Bianchi et al. 1970; del Moro et al. 1983a, b; Schaltegger et al. 2019). This is accompanied by an SW–NE gradient of increasing crustal contamination exemplified by incompatible elements (e.g., Rb, La; Dupuy et al. 1982; Macera et al. 1983), ^87^Sr/^86^Sr and δ^18^O (Cortecci et al. 1979; del Moro et al. 1983a, b), and decreasing εHf  in zircon (Schaltegger et al. 2019).

**Fig. 1:**
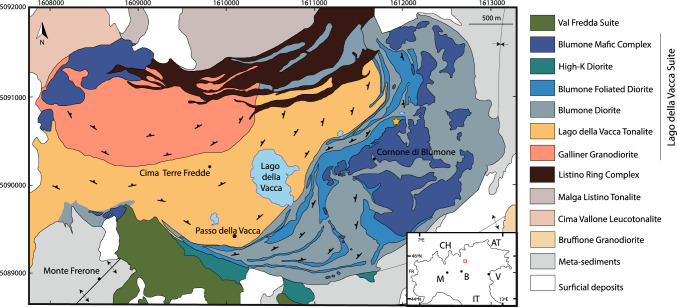
Simplified geological map of the Southern Re de Castello super-unit (modified after Verberne 2013) with sampling location of the Blumone nepheline-normative dykes (yellow star). The inset shows the location of the area in northern Italy. Contiguous countries and Italian cities are indicated for orientation (*M* Milano; *B* Brescia; *V* Venezia)

The Southern Re di Castello Unit (Fig. 1) is the southernmost unit of the Adamello batholith and is mainly composed of tonalites and granodiorites, and minor mafic intrusions (Bianchi et al. 1970). The Blumone complex is a volumetrically significant mafic complex, which was emplaced over a short time span of only ca. 50 kyr (Schoene et al. 2012). This complex can be subdivided into an ultramafic to mafic, vertically layered complex (Blumone s.s.) and a marginal unit, the Blumone intermediates (Brack 1983; Ulmer et al. 1983).

The Blumone mafic complex comprises plagioclase-bearing wehrlites, olivine-gabbros, amphibole gabbros, and clinopyroxene-bearing anorthosites. The main mafic lithology is a medium- to coarse-grained clinopyroxene-bearing to clinopyroxene-free amphibole gabbro (Colbertaldo 1940; Ulmer et al. 1983). Amphibole-poor varieties contain significant amounts of (low-Ti) magnetite (up to 10% locally; Ulmer 1986). The textural and lithological complexity supports the interpretation of a polyphase intrusive history, documented by grain size variation on a decimetre scale and irregular alternation of modally layered lithologies. There is also a wide variety of cumulate textures, from (olivine-)gabbro adcumulates, meso- to orthocumulate cpx-plag-magnetite gabbros with variable amounts of intercumulus hornblende, as well as local crescumulates (comb layers and very rarely orbicules). The mineralogy and textures suggest in situ accumulation of products crystallising from basaltic to basaltic–andesitic magmas (Ulmer et al. 1983). The Blumone intermediates range from diorites to quartz-diorites. Multiple cross-cutting relationships indicate that the intermediate unit was emplaced in multiple pulses (Ulmer 1986). The Blumone gabbroic complex has been interpreted as the root zone of an upper crustal magmatic–volcanic system where hot hydrous basaltic to basaltic–andesite magmas ascended from middle to lower crustal reservoirs (e.g., Ulmer et al. 1983; Ulmer 2007). The emplacement conditions were estimated at ca. 0.2 GPa, 1080–1150 °C, water-saturated, and fO_2_ around the NNO buffer (Ulmer 1986; Nimis and Ulmer 1998; Verberne 2013). The layered structure is often disrupted by younger injections of the Blumone intermediates.

## The Blumone nepheline-normative dykes

**Fig. 2:**
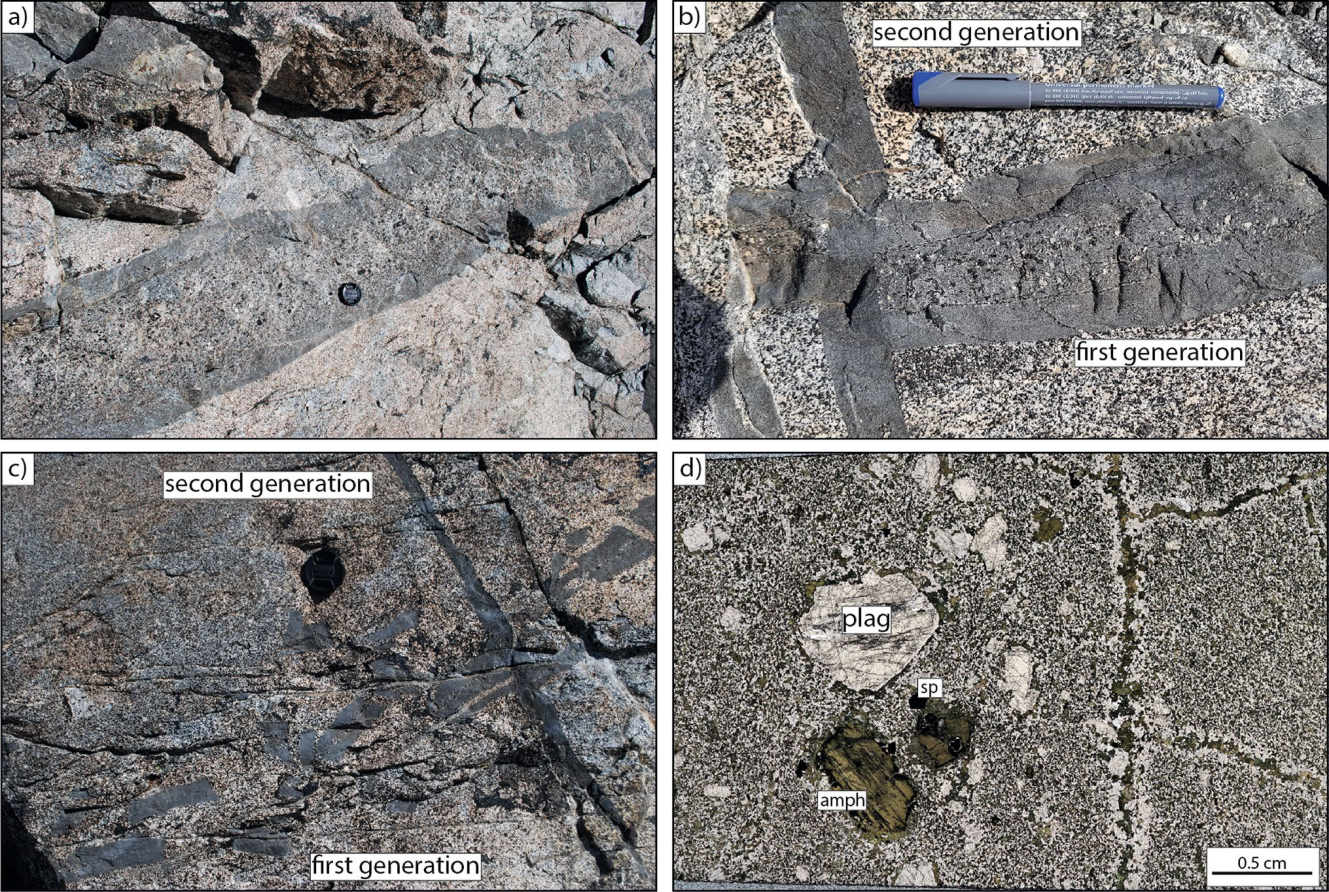
Field photographs and thin-section microphotograph documenting the Blumone ne-normative dykes’ emplacement features and crystal cargo. **a** First-generation dyke: thicker zones of the dykes exhibit a more significant proportion of crystal cargo, which is concentrated in the central part of the dyke. Chilled margins in contact to the host rocks are a common feature of these dykes. **b** Different cross-cutting dyke generations show different crystal cargo content and sharp contacts with the host rock. **c** Despite the sharp contact, some dykes are dismembered, especially the thinner ones. Lens cap for scale. **d** Phenocrysts of amphibole, plagioclase, and spinel are contained in a fine-grained matrix. Clinopyroxene is rare

Near the Blumone mafic complex (Fig. 1), a conspicuous dyke suite intrudes the Blumone intermediates (Fig. 2). Due to their anomalous geochemistry (i.e., their silica-undersaturated nature; see Bulk Rock Chemistry results section), which motivated the present study, we refer to them as the Blumone nepheline-normative (ne-normative) dykes. The dykes are divided into three generations in the field according to their structural orientation, cross-cutting relationships, and phenocryst presence (or absence).

The first generation of dykes has a width of approximately 20–50 cm and displays abundant phenocrysts (10–40 vol.%) of amphibole, clinopyroxene, plagioclase and spinel, and glomerocrysts of the same mineralogy concentrated in their central parts (Fig. 2a,b,d). The term phenocryst is used here, and in the rest of the manuscript, due to the difference in size between these crystals and the finer-grained matrix, with no implication of cognate link between such crystals and the dykes. The matrix is microphaneritic and composed of sub-millimetric amphibole, plagioclase, and Fe–Ti oxides (in order of decreasing abundance). The grain size is larger in the centre of the dyke than in the chilled margin. The narrower parts of these dykes are typically fragmented and contain less crystal cargo (Fig. 2c). The phenocrysts show distinct optical features compared to crystals in the matrix (pericline twinning in plagioclase crystals; brown colour of amphibole crystals).

The second-generation dykes intrude perpendicular to the first one, showing a maximum width of 10 cm. They are microphaneritic and typically trachytoid. The proportion of microphenocrysts is below 5 vol.%, mainly comprising plagioclase. The matrix comprises elongated and oriented plagioclase, amphibole, and Fe–Ti oxides (in order of decreasing abundance). Small and anhedral clinopyroxene inclusions can occasionally be observed in the hornblende crystals. The larger crystals comprise single plagioclase phenocrysts and hornblende–plagioclase glomerocrysts.

The third generation of dykes is cross-cutting the second one but exhibits similar features to the first generation.

## Methodology

### Analytical methods

#### Bulk rock geochemistry

The matrix of dyke samples was obtained by drilling with a 1 mm (inner diameter) micro-drill to ensure the absence of phenocrysts and subsequently crushed into a fine powder using a WC swing mill. An aliquot of this powder was heated for 90–120 min at 1050 °C in a ceramic crucible to determine the loss on ignition. The resulting powder was mixed with a Li-tetraborate and metaborate flux (1:5 ratio) and fused at 1080 °C. The resulting glass disks were analysed with a PANanalytical AXIOS wavelength-dispersive X-ray fluorescence (WD-XRF) spectrometer calibrated against in-house and international standards. The following major elements were analysed: Si, Ti, Fe, Mn, Mg, Ca, Na, K, and P.

Trace-element composition of the same bulk-rock samples was obtained by analysing small shards of broken glass disks by Laser Ablation–Inductively Coupled Plasma-Mass Spectrometry (LA–ICP-MS) at ETH Zürich. A GeoLas (Coherent, Germany) 193 nm Lambda Physik excimer ArF laser coupled to a PerkinElmer Nex-ION 2000 (Perkin-Elmer, USA/Canada) fast-scanning quadrupole ICP-MS was used. The samples were analysed with a spot size of 115 μm, repetition rate of 10 Hz, and laser energy density of 8–10 J cm^–2^. The analyses comprised ca. 30 s of gas blank acquisition and ca. 40 s of sample ablation. The carrier gas was composed of high-purity He (ca. 1 L/min) and Ar (ca. 1 L/min). The intensities of all isotopes were acquired with a dwell time of 10 ms. Calibration and drift correction were performed using NIST SRM610 synthetic glass (Jochum et al. 2011) as primary reference material (spot size of 40 μm) for calibration and instrumental sensitivity drift correction using the conventional standard–sample bracketing. An average of three measurements was taken for each sample. Drift correction and data reduction were done with the SILLS software (Guillong et al. 2008), using CaO contents (obtained by WD-XRF) as the internal standard for relative sensitivity correction. Isobaric interferences and potential contamination from the flux were corrected using a lithium meta- and tetraborate blank prepared and analysed following the same procedure as the samples, in which the apparent concentration was determined using Li as the internal standard. The accuracy and reproducibility of the analyses were checked using USGS BCR-2 as secondary reference material.

#### Phase chemistry

Thin sections of ne-normative dykes and Blumone cumulates, as well as experimental run products, were first examined with a JEOL JSM-6390 Scanning Electron Microscope (SEM) at ETH Zürich to characterise textural features at the microscopic level and select optimal spots for later quantitative analysis by electron microprobe. The SEM was also employed to examine the experimental products to assess equilibrium conditions and retrieve backscatter electron (BSE) images for the orientation of further EPMA and image analysis.

The major-element compositions of glass, plagioclase, clinopyroxene, olivine, amphibole, and spinel were analysed with a JEOL JXA 8230 Superprobe at ETH Zürich. An acceleration voltage of 15 kV was used for minerals and glasses, while CoPd alloys were measured with 20 kV. Selected standards, background and peak measurement times, and spectrometer assignment for the analysed elements were optimised for each phase. To minimise alkali migration, Na and K were analysed first for 10 s. Experimental glasses were analysed with a beam diameter of 20 μm and a current of 7 nA.

Trace-element concentrations of mineral phases (plagioclase, clinopyroxene, and amphibole) in natural samples were determined in thin sections by LA–ICP-MS at ETH Zürich, using a RESOlution (Australian Scientific Instruments) Excimer ArF (193 nm) laser ablation system coupled to an Element XR (ThermoFisher, Germany) sector-field ICP-MS. The samples and reference materials were analysed with a spot size of 29 or 43 μm, a repetition rate of 5 Hz, and a laser energy density of ca. 3.5 J cm^–2^. The carrier gas consisted of high-purity He (ca. 0.5 L/min) and Ar (ca. 1.0 L/min). The analyses consisted of 30 s gas blank acquisition followed by 30 s of sample ablation. Three pre-ablation pulses were performed for surface cleaning, and signal homogenisation was executed using in-house squid tubing. The ICP-MS instrument was optimised for maximum sensitivity of the high mass range, low oxide formation rate (ThO^+^/Th^+^ < 0.25%), and U/Th ratio of ca. 1 using the NIST SRM612 glass reference material. All elements were monitored for an equal dwell time of 10 ms. Drift correction and data reduction were performed using the SILLS software (Guillong et al. 2008). Al was used as the internal standard, with nominal concentrations determined by electron microprobe. The spot size varied between 9 and 43 μm depending on the crystal size and zoning pattern. NIST SRM 612 glass was used as the primary reference material for calibration and instrumental sensitivity drift correction using conventional standard-sample bracketing. BHVO-2G and GSD-1G (Jochum et al. 2005) were analysed as secondary reference materials. The typical analytical routine consisted of 2 NIST SRM 612, ca. 28 samples, 1 BHVO-2G, 1 GSD-1G, and 2 NIST SRM 612.

#### Sr isotope analysis

Strontium isotope compositions in plagioclase were determined by LA-MC–ICP-MS at ETH Zürich with an Australian Scientific Instruments RESOlution 193 nm ArF excimer laser probe connected to a Nu Instruments Plasma 2 multi-collector inductively coupled plasma mass spectrometer (MC–ICP-MS). Instrument tuning was undertaken by rastering NIST610 and a fragment of modern marine carbonate to optimise Sr signal intensity and peak shape. Oxide production rate was ≤ 0.2 wt.% based on the measurement of ThO^+^/Th^+^ in NIST610. For plagioclase analysis, laser conditions included a repetition rate of 5 Hz, a spot size of 100 μm, and an energy density of 4 J cm^−2^. Laser ablation was carried out in a He atmosphere with a flow rate of 350 ml min^−1^, and Ar was the carrier gas with N (2 ml min^−1^) added to increase sensitivity. Standards and samples were ablated for 40 s, preceded by 30 s of gas blank measurement, and followed by 30 s of sample washout. The following masses were monitored: 88, 87, 86.5, 86, 85.5, 85, 84.5, 84, 83.5, 83, 82.5, and 82. Kr corrections on masses 84 and 86 were performed with on-peak baseline correction. Mass bias was corrected using an exponential law and a reference ^88^Sr/^86^Sr ratio of 8.37520938 (Konter and Storm 2014). The intensity of ^85^Rb and a fixed ^87^Rb /^85^Rb of 0.385710 (Konter and Storm 2014) were used to correct the ^87^Rb interference on ^87^Sr. Ca dimer and argide interferences were monitored at mass 82 and 83 and corrected accordingly. The occurrence of doubly charged REE was monitored at the half masses, but no correction for isobaric interference was required due to negligible REE contributions. Data were reduced using the Iolite4 software (Woodhead et al. 2007; Paton et al. 2011).

Total Sr signals varied mainly between 0.7 and 1.1 V for both plagioclase reference materials (Thorn, Knob) and unknowns (Suppl. Table 3). Analytical accuracy and instrumental drift were evaluated by repeated ablation (every 8–10 measurements of plagioclase unknowns) of isotopically homogenous plagioclase phenocrysts (An_24-53_) separated from rhyolites from the Snake River Plain, USA: Thorn (primary reference material) and Knob (secondary) (Wolff et al. 2011). All data are reported relative to Thorn ^87^Sr/^86^Sr of 0.71306 (average TIMS data; Wolff et al. 2011) via standard bracketing. The weighted mean ^87^Sr/^86^Sr for Knob (0.70935 ± 0.00029, 2SE; *n* = 21; Supp. Table 3) is consistent with isotope TIMS and LA-MC-ICP-MS analyses of the same material (0.70939 ± 0.00002; Wolff et al. 2011). Mean ^84^Sr/^86^Sr of plagioclase reference materials and unknowns are within uncertainty of the natural ratio of 0.056511 ± 11 (Thirlwall 1991). ^87^Rb/^86^Sr ratios are negligible (i.e., generally < to <  < 0.001), which makes corrections for ^87^Sr/^86^Sr ingrowth insignificant. Therefore, the reported Sr isotope ratios are considered equal to the isotope ratios at the time of emplacement.

### Experimental methods

#### Starting material and pre-experimental procedure

The chilled margin of a dyke (B17-8), sampled in the upper Caffaro flank of Cornone di Blumone (see Fig. 1), was employed as starting material for the phase saturation experiments. The material is homogeneous under the optical and secondary electron microscope and contains no phenocrysts. The sample was ground in an agate mortar under ethanol to facilitate chemical equilibration during the experiments. Its bulk composition is presented in Table 1.

**Table 1:** Major- and trace-element chemistry, and normative mineralogy (after Grove 1993), of the ne-normative dykes

Generation	First				Second			Third
Samples	MP18-2	MP18-13	MP18-14	B-17-8^a^	MP18-1	MP18-12	B-17–9	MP18-11
*Major elements* (wt. %)								
SiO_2_	44.47	45.80	44.42	43.89	43.43	43.02	43.77	42.92
TiO_2_	1.27	1.22	1.32	1.39	1.32	1.34	1.20	1.25
Al_2_O_3_	20.41	20.18	20.23	20.31	20.55	20.53	22.02	21.42
Fe_2_O_3_	12.55	12.10	10.82	11.79	11.76	11.83	11.51	11.73
MnO	0.22	0.23	0.20	0.22	0.19	0.19	0.21	0.21
MgO	5.30	5.05	7.01	5.71	6.57	6.59	4.93	5.66
CaO	12.09	11.56	13.41	11.73	13.45	13.80	13.14	13.42
Na_2_O	2.35	2.87	1.87	2.45	1.69	1.71	2.23	1.84
K_2_O	0.33	0.18	0.33	0.18	0.33	0.23	0.25	0.23
P_2_O_5_	0.25	0.25	0.17	0.17	0.19	0.19	0.46	0.19
Total	99.24	99.43	99.79	97.84	99.46	99.42	99.71	98.86
xMg (FeO^tot^)	0.46	0.45	0.56	0.49	0.52	0.52	0.46	0.49
CaO/Al_2_O_3_ (mass)	0.59	0.57	0.66	0.58	0.65	0.67	0.60	0.63
*Grove (*1993*) normative mineralogy*^b^								
Quartz	-0.019	-0.014	-0.024	-0.025	-0.025	-0.034	-0.029	-0.031
Plagioclase	0.699	0.716	0.664	0.714	0.671	0.678	0.737	0.710
Olivine	0.144	0.136	0.146	0.146	0.151	0.147	0.132	0.141
Clinopyroxene	0.114	0.112	0.156	0.116	0.144	0.157	0.103	0.127
Ilmenite	0.018	0.017	0.018	0.020	0.018	0.019	0.017	0.018
Hematite	0.018	0.017	0.015	0.017	0.016	0.017	0.016	0.016
Orthoclase	0.021	0.011	0.021	0.011	0.021	0.014	0.016	0.014
Apatite	0.005	0.005	0.003	0.003	0.004	0.004	0.009	0.004
*Trace elements* (µg/g)								
Sc	24	22	40	33	31	33	24	29
V	391	386	417	272	413	416	308	365
Cr	8	5	54		6	54	8	7
Co	34	35	41		36	42	28	39
Ni	18	21	41		28	18	15	13
Zn	84	85	73		69	81	75	75
Ga	20	20	18		18	19	20	19
Rb	3.0	0.7	2.8	2.5	2.4	1.4	2.7	2.6
Sr	551	477	447	450	452	453	634	516
Y	22	21	17	29	15	16	24	20
Zr	15	24	29	25	22	23	27	23
Nb	4.0	4.0	4.0	4.2	3.3	3.4	4.2	4.6
Cs	0.17	0.05	0.18		0.09	0.11	0.11	0.10
Ba	69	72	58	67	54	47	58	64
La	13	13	7		6	6	13	9
Ce	33	29	20	42	18	17	29	23
Pr	4.4	3.8	2.8	5.4	2.6	2.6	4.1	3.3
Nd	18	17	13	23	12	12	19	15
Sm	4.3	4.0	3.3	5.5	3.1	3.4	4.6	3.6
Eu	1.4	1.3	1.2	1.8	1.2	1.2	1.4	1.3
Gd	3.7	3.8	3.3	5.6	2.7	3.2	4.7	3.5
Tb	0.6	0.6	0.5	0.9	0.5	0.5	0.7	0.6
Dy	4.0	3.7	3.3	5.6	2.9	3.2	4.5	3.5
Ho	0.8	0.8	0.6	1.2	0.6	0.7	0.9	0.7
Er	2.3	2.1	1.8	3.3	1.7	1.8	2.5	2.0
Tm	0.3	0.3	0.2	0.5	0.2	0.2	0.4	0.3
Yb	2.2	2.1	1.6	3.0	1.6	1.6	2.2	1.9
Lu	0.3	0.3	0.2	0.4	0.2	0.2	0.3	0.3
Hf	0.7	0.9	1.1	1.2	0.9	0.8	1.1	0.9
Ta	0.2	0.2	0.2		0.1	0.2	0.3	0.2
Pb	2.6	19.4	1.5	1.8	2.2	0.9	1.5	1.0
Th	0.4	0.1	0.4	0.3	0.2	0.1	0.6	0.2
U	0.20	0.04	0.09	0.06	0.06	0.07	0.15	0.08

^a^ The major-element chemistry of B-17–8 was obtained by EPMA analysis on a glass bead, resulting in lower total wt. %

^b^ Normative mineralogy (after Grove 1993) calculated with an Fe^3+^/Fe^tot^ = 0.2

Experiments were performed in Au_90_Pd_10_ capsules (Au at 975 °C) with outer and inner diameters of 2.3 and 2.0 mm, respectively. The capsules were loaded with 20–25 mg of starting material and 5 wt.% deionised H_2_O to achieve water-saturated conditions, sealed using a Lampert PUK U4 welder and submerged in acetone overnight to assess potential leaks.

Evidence from the southern Adamello batholith strongly supports water-saturated conditions during magmatic activity. The intrusion of the batholith occurred at 200–300 MPa (e.g., Nimis and Ulmer 1998), where the solubility of H_2_O in the silicate melt is much higher than CO_2_ (e.g., Papale 1999). Southern Adamello mafic rock mineral chemistry and fluid inclusion studies confirm the presence of miarolitic cavities (indicating fluid saturation) and H_2_O abundance relative to CO_2_ in the fluid inclusions (Ulmer 1986; Hennings et al. 2017). Recent research affirms the superhydrous nature (above 8 wt. % H_2_O) of Adamello primary magmas (Müntener et al. 2021), and, thus, the dominating role of H_2_O in the volatile budget during differentiation. Fluid-saturated conditions are further supported by the widespread occurrence of gabbroic pegmatoids and hydrofracturing within the gabbroic complex.

#### Experimental apparatus

Phase equilibria experiments were performed in rapid-quench externally heated HCM (Hf-C-Mo alloy) pressure vessels at ETH Zürich. A mixture of argon and methane was used as the pressure medium. Methane was used as a reagent component to buffer the hydrogen fugacity (fH_2_) and, thus, oxygen fugacity (fO_2_) close to the Ni–NiO (NNO) equilibrium. Since the experiments were performed under water-saturated conditions, the fO_2_ of the system was buffered at NNO, representing conditions close to the lower limit inferred for typical arc-related calc-alkaline magmas (Carmichael 1991; Cottrell et al. 2022). The experiment started with vessel pressurisation at room temperature with half of the intended run pressure, and final pressures were reached via gas expansion upon heating. The limitations of this approach in precisely controlling the experimental fH_2_ have been discussed by Marxer and Ulmer (2019) and Alex and Zajacz (2020). We applied a similar approach to monitor experimental fO_2_ employing CoPd redox sensors. The calibration of Marxer and Ulmer (2019) for the Co–Pd–CoO–H_2_O system was utilised, following Taylor et al. (1992), with a pressure correction term by Pownceby and O’Neill (2000) and the expression of dG_Co-CoO_ of O’Neill and Pownceby (1993).

Experiments were performed from 975 to 1100 °C, with 25 °C intervals. Temperatures were monitored with K-type thermocouples. Tilting the vessel by 10° minimised temperature gradients. 2σ errors on the calibrated temperature are below 10 °C. Experimental pressure shows a 1 MPa maximum deviation at 200 MPa. Run durations were selected to attain a close approach to chemical equilibrium while minimising iron loss to the AuPd capsule and hydrogen loss from the capsules and vessel to maintain constant fO_2_ during the experiment. Quenching was performed by vertically tilting the vessel, forcing the capsules to drop to the cold end resulting in cooling rates exceeding 100 °C/s.

Recovered capsules were weighed after the experiment to assess potential water loss. Successful charges were mounted in epoxy resin and ground longitudinally to expose a middle section of the charge and polished with diamond suspensions down to 1 μm.

### Thermal model setup

To simulate the thermal evolution and quantify conditions necessary for cumulate melting, we performed one-dimensional model calculations of the lateral temperature evolution of a cumulate pile subjected to subsequent vertical dyke injections where dykes act as conduits accommodating some magma flow following emplacement. The latter effect is captured by holding the dyke temperature constant for a finite time. The model is illustrated in Figure S1. The 1D model domain represents a horizontal cross-section through a cumulate pile assumed to be more extensive and uniform in the vertical and second horizontal direction. The simulations test two parameters over a wide range: the number of injections and the time each dyke accommodates magma flow through the cumulate pile upon emplacement.

Given that our model focuses on the thermal effects of dykes acting as conduits for a finite duration in a horizontal 1D domain of cumulates, our approach follows that of Tornare et al. (2018). This approach differs from the works of Annen and Sparks (2002) and Dufek and Bergantz (2005), who focus on the thermal relaxation around instantaneously emplaced sills in 1D vertical crustal sections.

Thermal evolution follows from the conservation of energy:

1 $$\frac{\partial }{\partial t}\left( {\rho C_{p} T + \rho \varphi L} \right) = \nabla \cdot k\nabla T,$$

where $\rho$ is density [kg m^−3^], ${C}_{p}$ is specific heat capacity [J kg^−1^ K^−1^], T is temperature [K], $\varphi$ is volumetric melt fraction, $L$ is latent heat [J kg^−1^], and $k$ is thermal diffusivity [m^2^ s^−1^]. We assume constant density (2950 kg m^−3^), heat capacity (1500 J kg^−1^ K^−1^), and thermal diffusivity (10^–6^ m^2^ s^−1^). We consider a linear variation of the latent heat (4.0E + 5 J kg^−1^) between the liquidus (1110 °C) and solidus (850 °C) temperatures. The liquidus temperature was chosen based on the experiments of Marxer et al. (2023) on a hydrous arc basalt at 2 kbar. The choice of solidus temperature was based on the T–f trajectory of their experiments and is within the range of the estimates by Baker and Eggler (1983) and Holloway and Burnham (1972).

We employ an equilibrium approach where melt fractions are an instantaneous function of temperature. The melt fraction–temperature relationship (Figure S2) follows a linear fit of the experimental basaltic equilibrium crystallisation series at 200 MPa by Marxer et al. (2023). With these simplifying assumptions, Eq. (1) reduces to

2 $$\rho C_{p} \frac{\partial T}{{\partial t}} + \rho L\frac{\partial \varphi }{{\partial t}} = k\frac{{\partial^{2} T}}{{\partial x^{2} }}.$$

Equation (2) is discretised using a staggered grid finite difference scheme and integrated with explicit time stepping. A Picard–Lindelöf iteration scheme is used to resolve the non-linearity between temperature, melt fraction, and latent heat. Convergence is considered to be achieved when the temperature residual between two consecutive iterations falls below a defined tolerance.

The horizontal domain size is 10 km, and the nodes were spaced 0.5 m. The duration of each simulation is set to 50 ky, as constrained by high-resolution U–Pb zircon dating of the Blumone cumulate and intermediates intrusions (Schoene et al. 2012). To minimise the computational effort, the time step is set to a constant value chosen to ensure numerical accuracy and respect the Courant–Friedrichs–Lewy condition. The following adaptative Dirichlet boundary conditions are applied on either end of the horizontal 1D domain:

3a $$T_{x = 0} = \frac{{{\Delta }x}}{\omega }T_{x = - \infty } + \left( {1 - \frac{{{\Delta }x}}{\omega }} \right)T_{{x = {\Delta }x}}$$

3b $$T_{{x = x_{max} }} = \frac{{{\Delta }x}}{\omega }T_{x = + \infty } + \left( {1 - \frac{{{\Delta }x}}{\omega }} \right)T_{{x = x_{max} - {\Delta }x}} ,$$

where $\Delta x$ corresponds to the node spacing. We assume the wall rock is thermally buffered from the modelled cumulate pile at a given distance. $\omega$ corresponds to the distance to the assumed far-field isotherm, and ${T}_{x=\pm \infty }$ correspond to the temperature of the far-field isotherm. We consider the far-field isotherm to be equal to the wall rock temperature.

The horizontal model initially considers 2 km of partly crystallised gabbroic cumulates at T = 950 °C, surrounded by host rock at T = 240 °C. In an active magmatic arc system, such a high temperature can be expected at ca. 200 MPa (e.g., Blundy and Holland 1990).

We vary two parameters in the simulations to assess their effect on cumulate melt production and the thermal evolution of the cumulate system: the number of injections and the flow time. The number of injections is varied from 25 (40 m) to 250 (4 m), resulting in the same final magma injection width of 1 km. These injections are emplaced regularly during 50 kyr of simulation. As a result, a higher total injection number results in a higher intrusion frequency with lower injection width. Injections are emplaced randomly in the central 600 m cumulate zone with an injection temperature of 1070 °C corresponding to the liquidus temperature of a basaltic andesite bracketed by the experiments of Martel et al. (1999) and Marxer et al. (2023). We use the same notion of flow time as Floess & Baumgartner (2015) and Tornare et al. (2018), such that a dyke maintains the injection temperature for a specified duration. The flow time is varied between 1 and 50 years. Natural magmatic feeding systems likely exhibit intermittent flow, similar to what is observed at open-conduit volcanoes. However, resolving such complex behaviour would not add to the understanding gained from our purposefully simple model. Hence, flow time here specifically refers to the time over which a dyke is held at injection temperature regardless of whether that might be justified by assuming continuous magma flow or intermittent pulses of sufficiently fast succession that the conduit is not allowed to solidify in between. Volumetric flow rates are calculated based on the equation for Poiseuille flow by Delaney and Pollard (1982). Details can be found in the supplementary material.

Observations in the Blumone area show a range of dyke widths consistent with our input parameters. In addition, our choice of the range of tested injection widths falls within reasonable limits considering the dyke width compilation of Wada (1994). Although we did not observe pulses wider than 40 m in the Southern Adamello complex, this upper limit allows to test the efficiency of cumulate melting under more extreme conditions in other batholiths, rather than for our specific setting. The parameter range tested for flow times is more challenging to constrain from observations. Here, we select a wide range from a low value near the characteristic time of diffusive cooling of the narrowest dykes we model to a high value representing systems with substantial magma fluxes.

## Results

### Bulk rock chemistry

**Fig. 3:**
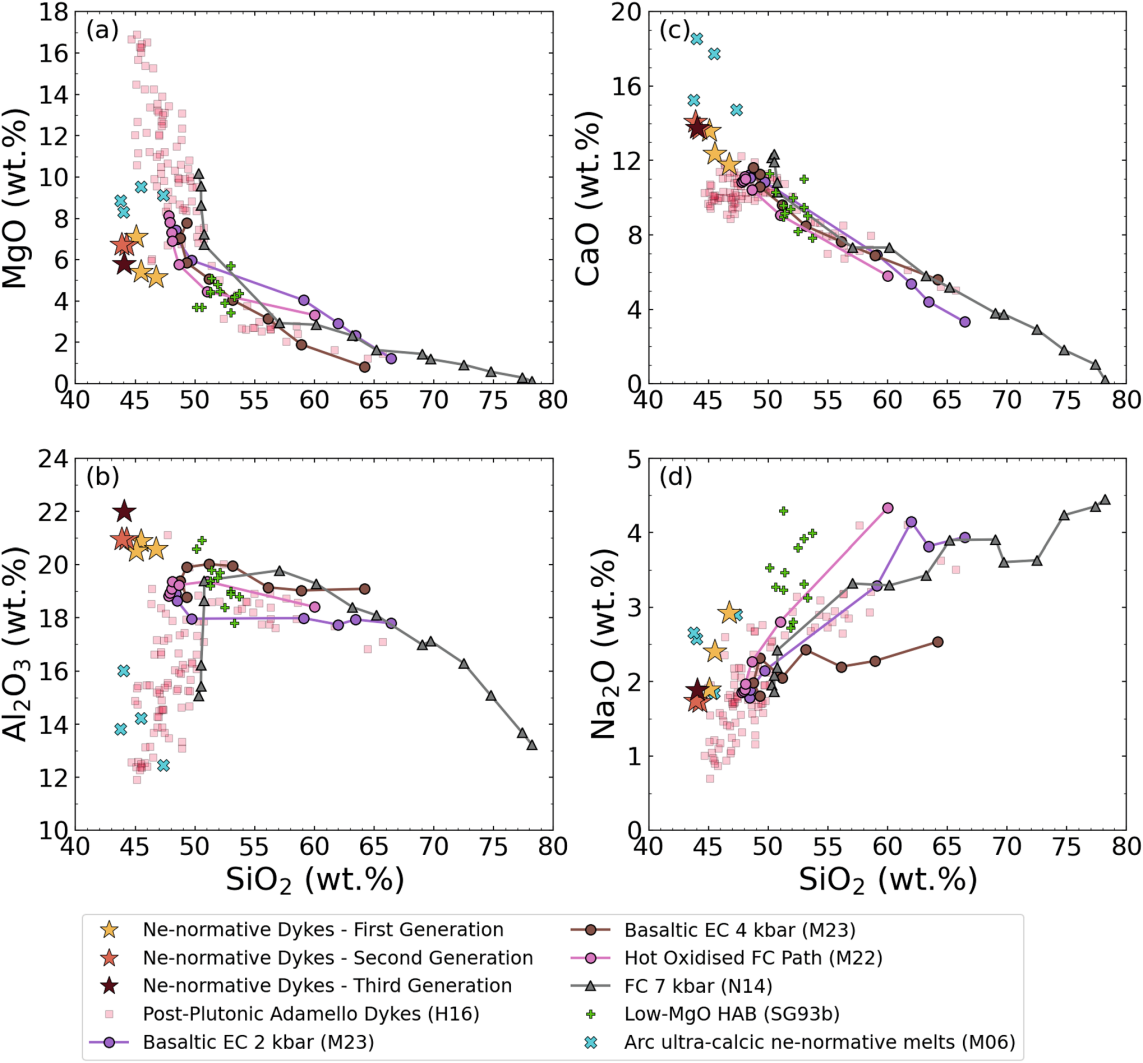
Variation diagrams of selected major oxides (in wt.%) as a function of SiO_2_ (wt.%) of the nepheline-normative dykes. In addition, relevant experimentally determined liquid lines of descent are plotted with representative compositions of the southern Adamello batholith, low-MgO high-alumina basalts, and ultra-calcic, nepheline-normative melts. H16: Hürlimann et al. (2016); M23: Marxer et al. (2023); M22: Marxer et al. (2022); N14: Nandedkar et al. (2014); SG93b: Sisson & Grove (1993b); M06: Médard et al. (2006). The following abbreviations were used: *EC* equilibrium crystallisation, *FC* fractional crystallisation, *HAB* high-alumina basalt

Analyses of the Blumone dykes are reported in Table 1 and illustrated in Fig. 3, together with dyke compositions of the Adamello batholith (representative of the general compositional spread of arc magmas; Hürlimann et al. 2016), compiled high-alumina basalt compositions (Sisson and Grove 1993b), ultra-calcic, nepheline-normative melts (Médard et al. 2006), and relevant experimental data (Nandedkar et al. 2014; Marxer et al. 2022, 2023). The dykes are ne-normative and have distinct chemical features compared to calc-alkaline rocks (Fig. 3) expressed by their high concentrations of Al_2_O_3_, CaO, Na_2_O and FeO, and low MgO and Mg# at given (low) SiO_2_.

**Fig. 4:**
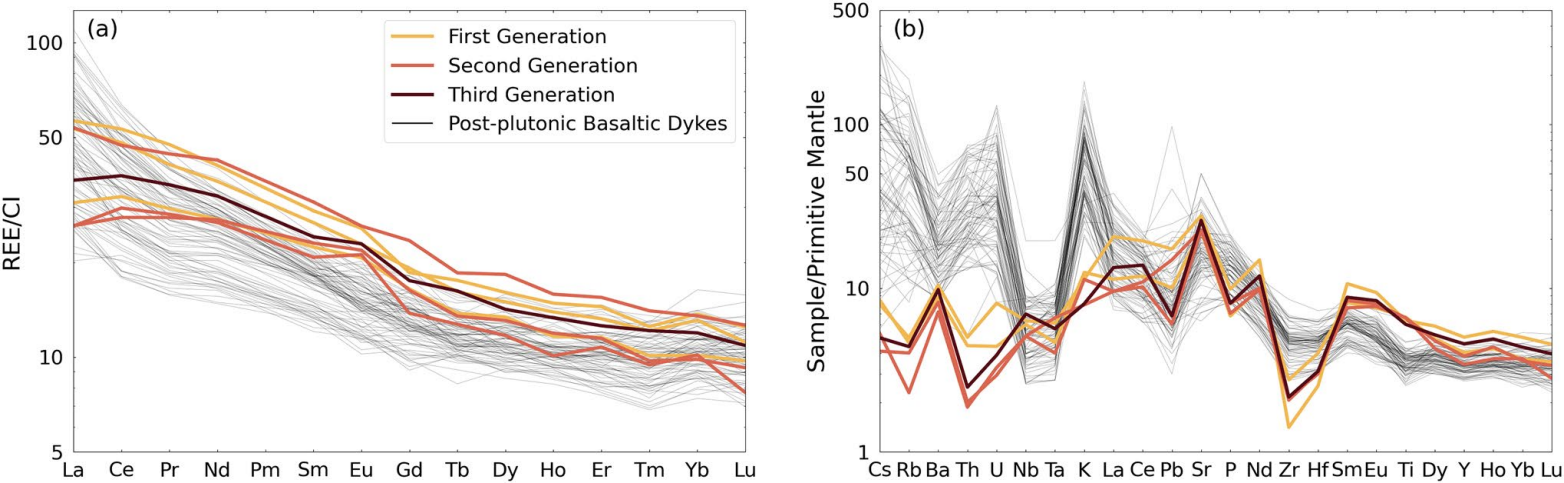
The **a** Chondrite-normalised (McDonough and Sun 1995) rare-earth element concentrations and **b** primitive mantle-normalised (McDonough and Sun 1995) trace-element contents of the ne-normative dykes and Southern Adamello post-plutonic basaltic-to-dacitic dykes (Hürlimann et al. 2016)

The different dyke generations exhibit minor differences in terms of whole-rock chemistry. Noteworthy are the higher SiO_2_ (43.9–45.8 wt.%), Na_2_O (1.9–2.7 wt.%), and lower CaO contents (11.5–13.4 wt.%) of the first generation. However, we observe some scatter in Mg#. Chondrite-normalised REE patterns reveal a pronounced LREE enrichment over HREE ranging from 2.6 ± 0.6 to 3.7 ± 0.6 (Fig. 4a). The MREE-to-HREE patterns are steeper than for calc-alkaline basalts to dacites (Fig. 4a; Hürlimann et al. 2016), with (Dy/Yb)_N_ ranging from 1.14 to 1.33. A modest positive Eu anomaly can be observed. The primitive mantle-normalised spider diagram (Fig. 4b) displays atypical features compared to continental arc calc-alkaline magmas (e.g., Hürlimann et al. 2016), such as the distinctly lower LILE contents. The first generation exhibits a slightly more enriched REE pattern and higher normalised Sr and Ba contents.

### Mineral chemistry, Sr isotopes, and thermometry

#### Phenocrystic and cumulate plagioclase

**Fig. 5:**
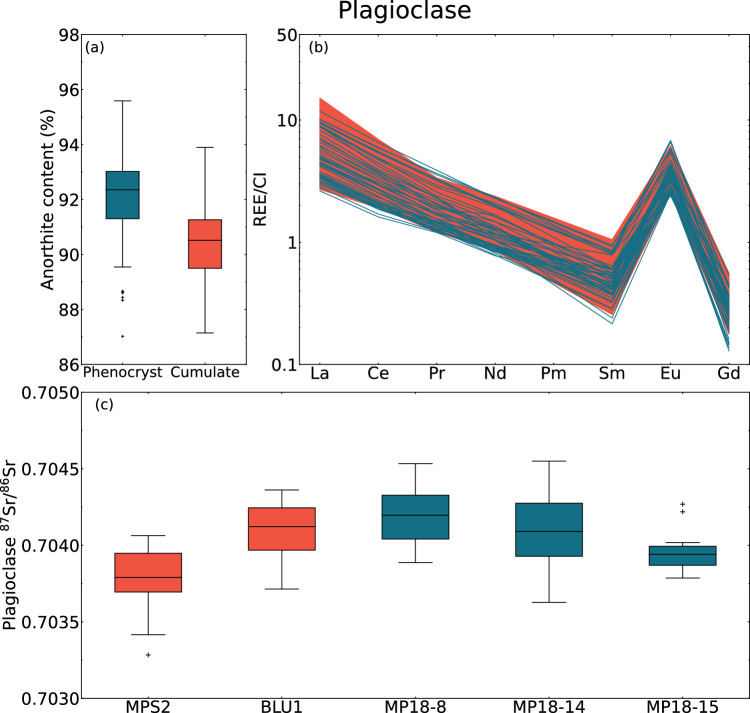
Comparison of chemical characteristics of phenocrystic (blue, *n* = 58) and cumulate (red, *n* = 88) plagioclase. **a** Anorthite content. **b** Chondrite-normalised (McDonough & Sun 1995) light-to-medium rare-earth element contents. **c** ^87^Sr/^86^Sr ratio of plagioclase phenocrysts and cumulate crystals, respectively

The cores of the phenocrystic plagioclase in the ne-normative dykes show several chemical features common with cumulate plagioclase of the adjacent Blumone mafic complex. Although the phenocrystic plagioclase is slightly An-richer, both populations are unimodal and mostly An_>90_ (Fig. 5a). This indicates that both plagioclase populations can be ascribed to similar, and early, stages of differentiation of a hydrous basalt at low pressure (e.g., Arculus and Wills 1980; Panjasawatwong et al. 1995). In addition, some of the cumulates measured are olivine-bearing adcumulates, whose plagioclase anorthite content is slightly higher than for amphibole gabbros, reconciling the misfit. This is further supported by the overlapping LREE patterns of both groups (Fig. 5b).

The Sr isotopic composition of plagioclase crystals in two samples of nepheline-normative dykes and two cumulate rocks is homogeneous and shows similar ^87^Sr/^86^Sr values (i.e., cumulates: BLU1–0.70410 ± 8 (2se; *n* = 22), MPS2–0.70382 ± 7 (*n* = 18); ne-normative dykes: MP18-14–0.7041 ± 2 (*n* = 10), MP18-15–0.7040 ± 2 (*n* = 11), MP18-8–0.7042 ± 1 (*n* = 14); Fig. 5c; Supplementary File S3). These values are similar to the whole-rock Sr isotopic signature of different Blumone lithologies (0.70412–0.70415; Kagami et al. 1991) and post-plutonic high-MgO basalts (Hürlimann et al. 2016).

Clinopyroxene and amphibole mineral data further support the close genetic relationship between the Blumone cumulates and the ne-normative dykes (information provided in the supplementary text and Figures S3 and S4, including the mineral chemistry of the matrix amphibole and plagioclase of the ne-normative dykes, in Figure S5).

#### Amphibole–plagioclase thermometry

**Fig. 6:**
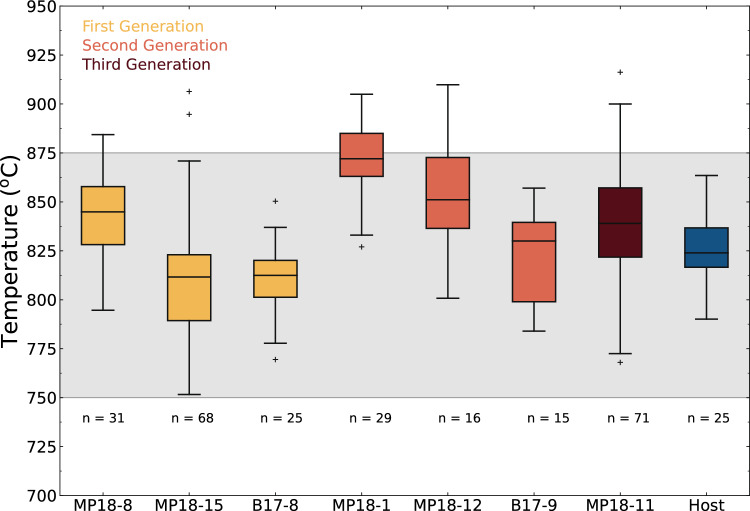
Results of the amphibole-plagioclase (edenite-richterite; Holland and Blundy 1994) equilibration temperatures in the dykes, discriminated by subgroup, and host quartz–diorite (blue). The grey band represents the temperature range of a dioritic–tonalitic system with 35–50 wt.% melt (Marxer and Ulmer 2019)

Amphibole–plagioclase equilibration temperatures based on the matrix minerals of the ne-normative dykes were calculated at 200 MPa (e.g., Nimis and Ulmer 1998) using the edenite + albite = richterite + anorthite equilibrium (Holland and Blundy 1994) (for details, see supplementary text). The results are shown in Fig. 6. The average equilibration temperatures of the three dyke generations overlap: 821 ± 41 °C (1st generation; N = 3), 841 ± 41 °C (2nd generation; N = 3), and 837 ± 30 °C (3rd generation; N = 1). The recorded temperatures correspond to equilibration with the surrounding quartz-dioritic host rock, as shown by the similar equilibration temperature calculated in the host rock (blue boxplot in Fig. 6).

### Experimentally determined phase equilibria

#### General remarks

**Fig. 7:**
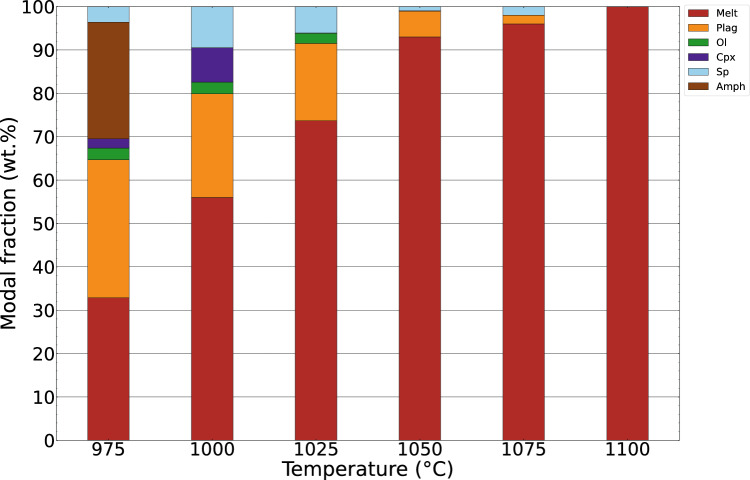
Modal phase proportions (wt.%) of the experimental runs as a function of temperature

Several observations infer that chemical equilibration was closely approached in our experiments: (1) The high melt fraction (Fig. 7, Table 2; Figure S6) and, thus, melt interconnectivity promote the chemical interaction of the crystallising phases. (2) All phases display, within analytical error, homogeneous compositions (Table 3). (3) Most phases are idiomorphic and chemically homogeneous, with some notable exceptions. Relict amphibole cores are observed at 975 °C. However, these are volumetrically minor (< 20% of total crystal) and do not significantly influence phase equilibria. Olivine is typically zoned, owing to continuous Fe loss (maximum 6.6 wt.% in olivine-bearing experiments) to the Au_90_Pd_10_ capsule, resulting in a forsterite-enriched rim. At 1000 °C, clinopyroxene exhibits sector zoning in Mg# and Al_2_O_3_, most likely related to rapid pyroxene growth (Schwandt & Mckay 2006; Nandedkar et al. 2014). (4) Mineral melt distribution coefficients agree with the previous studies (Table 3). The high Ca–Na distribution coefficient between plagioclase and melt (4.2 at 1075 °C and above 5.5 for the lower T experiments) suggests equilibration under H_2_O-saturated conditions (Sisson and Grove 1993a). The calculated amphibole-melt K_D_^Fe−Mg^ at 975 °C (0.31) overlaps the range 0.30–0.38 of Sisson and Grove (1993a). Experimental olivine at 1000 °C exhibits a rim K_D_^Fe−Mg^ of 0.26; a similar value is observed at 975 °C, within the range of experimental studies (e.g., Nandedkar et al. (2014) 0.24–0.31; Ulmer et al. (2018)  – 0.22 to 0.26), consistent with fO_2_ conditions around NNO (Ulmer 1982). The calculated clinopyroxene K_D_^Fe−Mg^ are higher for the high-Al than for the low-Al sectors, in accordance with Nandedkar et al. (2014).

**Table 2:** Summary of experimental run conditions

Run	T (°C)	*t*(*h*)	Capsule	Run products^a^	Phase proportions (wt.%)^b^	Fe loss (%)^c^	∑R^2 d^	Melt H_2_O (wt.%)^e^	fO_2_(∆NNO)^f^
MS125	975	120	Au	Melt, ol, cpx, amph, plag, sp	32.9(5); 2.7(5); 2.1(6); 27(2); 31.8(6); 3.6	0.09	0.009	5.4	0.5(2)
MS1	1000	48	Au_90_Pd_10_	Melt, ol, cpx, plag, sp	56.0(3); 2.7(1); 7.9(2); 23.9(2); 9.5	–	0.017	5.6	–
MS40	1025	48	Au_90_Pd_10_	Melt, ol, plag, sp	73.7(8); 17.8(6); 2.4(3); 6.1	6.57	0.044	5.2	0.2(1)
MS30	1050	24	Au_90_Pd_10_	Melt, plag, sp	93(1); 6(1); 0.7(4)	10	0.08	5.7	0.1(1)
MS37	1075	4	Au_90_Pd_10_	Melt, plag, sp	96(2); 2(2); 2	8	0.3	4.6	– 0.1(1)
MS40	1100	4	Au_90_Pd_10_	Melt	100	15		–	0.0(2)

^a^Identified stable phase assemblage. Abbreviations for phases: *plag *plagioclase, *cpx *clinopyroxene, *amph *amphibole, *sp* spinel

^b^Phase proportions calculated by mass balance regression. Sp proportions were estimated from BSE images of run charges. Numbers in parentheses read like 32.9(5) = 32.9 ± 0.5

^c^Fe-loss calculated by mass balance regression using a fictive FeO phase and expressed relative to the FeO content of the starting material

^d^Sum of the squared residuals from mass balance regression

^e^Melt water content (wt. %) estimated by plagioclase-melt hygrometry (Waters and Lange 2015)

^f^Oxygen fugacity measured with CoPd alloys; CoPd sensor was not used in MS1 run

**Table 3:** Average major-element composition of experimental phases (wt. %) determined by EPMA. The standard deviation is given in the second line of each phase

Run	T (ºC)	Phase	#^a^	SiO_2_ (wt.%)	TiO_2_ (wt.%)	Al_2_O_3_ (wt.%)	FeO (wt.%)	MnO (wt.%)	MgO (wt.%)	CaO (wt.%)	Na_2_O (wt.%)	K_2_O (wt.%)	P_2_O_5_ (wt.%)	Total^b^	An content	xMg	Kd Ca–Na	Kd Fe–Mg
MS47	1100	Melt	21	46.3	1.28	21.3	10.3	0.21	5.50	12.1	2.6	0.18	0.24	93.7		0.489		
				0.2	0.06	0.1	0.1	0.02	0.08	0.2	0.1	0.01	0.03	0.4		0.009		
MS37	1075	Melt	23	45.8	1.36	20.0	11.6	0.25	6.0	11.8	2.76	0.20	0.27	93.5		0.48		
				0.3	0.05	0.2	0.2	0.03	0.1	0.2	0.10	0.01	0.04	0.5		0.01		
		Plag	17	45.2	0.02	35.0	0.7	b.d.^c^	0.07	18.3	1.0	0.015		100.3	0.91		4.2	
				0.3	0.01	0.2	0.1		0.03	0.2	0.1	0.004		0.3	0.01		0.3	
		Spinel	22	b.d.^c^	0.5	54	26	0.19	15.8	0.17	b.d.^c^	b.d.^c^	b.d.^c^	96.7		0.52		
					0.1	1	1	0.02	0.4	0.05				0.3		0.01		
MS30	1050	Melt	22	45.9	1.38	19.8	11.5	0.24	6.07	11.7	2.8	0.20	0.26	92.2		0.484		
				0.2	0.08	0.1	0.1	0.03	0.08	0.1	0.1	0.01	0.03	0.4		0.009		
		Plag	17	44.5	0.03	35.2	0.7	b.d	0.06	18.6	0.8	0.015	b.d	99.9	0.93		5.6	
				0.4	0.02	0.3	0.1		0.03	0.3	0.2	0.004		0.3	0.02		0.8	
		Spinel	21	b.d.^c^	0.43	55	24.5	0.18	16.0	0.16	0.02	b.d	b.d	96.6		0.54		
					0.05	1	0.6	0.02	0.3	0.07	0.01			0.6		0.02		
MS40	1025	Melt	18	49.4	1.35	19.0	8.5	0.26	5.89	11.8	3.17	0.24	0.32	92.9		0.55		
				0.2	0.07	0.1	0.1	0.03	0.09	0.2	0.09	0.02	0.05	0.3		0.01		
		Plag	17	45.0	0.02	35.6	0.6	b.d.^c^	b.d.^c^	0.067	19	0.8	b.d.^c^	100.9	0.93			
				0.5	0.02	0.5	0.1			0.002	4	0.6		0.5	0.01			
		Ol core	14	39.2	0.03	0.04	18	0.42	41	0.37	b.d.^c^	b.d.^c^	b.d.^c^	99.2		0.80		0.31
				0.3	0.02	0.02	1	0.01	1	0.03				0.4		0.02		0.01
		Ol rim	8	39.9	0.04	0.13	14.9	0.41	43.4	0.38	b.d.^c^	0.010	b.d.^c^	99.3		0.838		0.24
				0.1	0.02	0.08	0.5	0.01	0.8	0.03		0.005		0.4		0.007		0.02
		Spinel	23	0.1	4.53	11	71	0.40	7.3	0.07	b.d.^c^	b.d.^c^	b.d.^c^	94.5		0.155		
				0.0	0.49	1	1	0.03	0.2	0.04				0.5		0.007		
MS1	1000	Melt	26	51.5	1.32	19.4	7.5	0.28	5.10	10.2	4.0	0.31	0.42	92.7		0.55		
				0.2	0.08	0.1	0.1	0.04	0.06	0.1	0.1	0.01	0.04	0.3		0.01		
		Plag	22	45.3	0.04	35.3	0.7	b.d.^c^	b.d.^c^	18.5	0.9	0.011		101.0	0.92		7.9	
				0.5	0.05	0.4	0.2			0.4	0.2	0.008		0.5	0.02		0.6	
		Low-Al cpx	12	50.7	0.78	4.3	6.8	0.24	14.9	22.3	0.26	b.d.^c^	0.017	100.2		0.80		0.31
				0.5	0.07	0.3	0.4	0.01	0.3	0.2	0.02		0.009	0.2		0.01		0.01
		High-Al cpx	18	46	1.7	8.3	8.3	0.19	12.3	22.6	0.33	b.d.^c^	0.016	99.8		0.73		0.46
				1	0.3	0.8	0.4	0.02	0.5	0.2	0.02		0.008	0.3		0.02		0.03
		Ol core	17	38.9	0.05	0.036	19	0.49	40	0.35	b.d.^c^	b.d.^c^	0.11	99.4		0.79		0.32
				0.3	0.02	0.009	1	0.02	1	0.02			0.06	0.3		0.02		0.02
		Ol rim	11	39.6	0.04	0.08	16.3	0.49	42.7	0.37	b.d.^c^	b.d.^c^	0.04	99.6		0.82		0.26
				0.3	0.02	0.03	0.9	0.02	0.8	0.02			0.03	0.4		0.01		0.02
		Spinel	28	0.10	5.0	8.5	73.5	0.51	7.2	0.16	b.d.^c^	b.d.^c^	b.d	95.2		0.15		
				0.01	0.4	0.2	0.6	0.03	0.1	0.06				0.4		0.02		
MS125	975	Melt	20	52.6	0.92	18.3	10.6	0.32	3.37	8.7	4.3	0.38	0.63	91.1		0.361		
				0.2	0.07	0.1	0.2	0.04	0.07	0.1	0.2	0.03	0.05	0.5		0.008		
		Plag	15	45.9	b.d.^c^	34.2	0.70	0.02	0.04	17.5	1.3	0.013	0.01	99.8	0.88		6.5	
				0.3		0.3	0.07	0.01	0.01	0.3	0.1	0.008	0.01	0.3	0.02		0.4	
		Ol	10	37.3	0.03	0.02	27	0.76	33	0.25	0.01	b.d.^c^	0.05	99.0		0.67		0.26
				0.7	0.02	0.01	3	0.03	2	0.03	0.01		0.02	0.5		0.04		0.02
		Amph relic	8	44.4	1.8	10.8	12.8	0.40	13.0	11.5	2.0	0.34	b.d.^c^	96.9		0.64		0.31
				0.4	0.2	0.4	0.2	0.03	0.3	0.2	0.2	0.03		0.3		0.01		0.02
		Amph	17	40.8	2.7	13.4	12.5	0.20	12.7	11.6	2.74	0.15	b.d.^c^	96.9		0.64		0.31
				0.4	0.1	0.2	0.5	0.02	0.5	0.2	0.07	0.01		0.4		0.02		0.02
		Cpx	3	48.6	1.0	6.1	10.3	0.31	11.7	21.0	0.5	b.d.^c^	b.d.^c^	99.5		0.67		0.28
				0.7	0.2	0.4	0.7	0.02	0.3	0.9	0.2			0.3		0.02		0.01
		Sp	22	0.10	8	5.7	76	0.47	2.9	0.18	b.d.^c^	b.d.^c^	0.010	93.7				
				0.02	2	0.2	2	0.04	0.2	0.05			0.007	0.3				

All Fe as FeO. Units in the second line correspond to the standard deviation of averaged analyses

^a^ Number of individual analyses per phase

^b^ Melt composition is normalised to 100 wt.%, but EPMA non-normalised totals are reported

^c^ Below detection limit

#### Phase relations and proportions

Phase proportions of experimental products were estimated with the non-weighted, least-square regression algorithm implemented in Microsoft Office Excel based on the measured phase chemistry (Table 3). The spinel proportion was estimated from BSE images and converted into mass proportion by considering the starting material mass and the density of each phase. Densities of mineral phases were estimated based on Abers and Hacker (2016); melt density was calculated with the algorithm of Iacovino and Till (2019) and references therein.

Calculated experimental phase proportions are illustrated in Fig. 7. The liquidus temperature is located between 1075 and 1100 °C. Melt proportions decrease non-linearly with decreasing temperature. The decrease is strongly enhanced below 1050 °C, coinciding with the saturation of Fe–Mg silicate phases. Plagioclase and hercynitic spinel saturate at near-liquidus conditions, as documented in the 1050 and 1075 °C experiments. At 1025 °C, olivine is stable, and the spinel (aluminous titano-magnetite) and plagioclase proportions increase. Clinopyroxene saturates between 1025 and 1000 °C, accompanied by an increase in the proportions of spinel and plagioclase. The saturation of amphibole between 1000 and 975 °C results in a pronounced decrease in spinel and clinopyroxene proportions and coincides with an increase in the plagioclase fraction; the olivine mass fraction remains constant, although with a decrease in the forsterite content. The lower proportions of spinel and clinopyroxene at 975 with respect to 1000 °C indicate their role as peritectic reactants in the amphibole-forming reaction. The decrease in the spinel fraction leads to an increase in the melt FeO content (Figure S7) and, therefore, decrease in xMg.

The mineral chemistry of the experimental products is given in Table 3, and a detailed description of phase compositions is provided in the supplementary text.

### Thermal modelling

The thermal model simulations assess the evolution of the proportion of cumulate melting across the 1D horizontal section as a function of dyke width and flow time of melt injections. A key metric extracted from the model results is the proportion of the cumulate pile heated to temperatures exceeding 1050 °C. This is the lowest experimental temperature where ne-normative melt was observed in equilibrium with plagioclase and spinel (Fig. 7). In the following, we refer to the parts of the cumulate pile heated to above 1050 °C as high-temperature cumulate melts. In contrast, we refer to the proportion of the cumulate pile heated to above 850 °C as suprasolidus cumulates. The latter is a second key metric indicating how much of the modelled cumulate pile has experienced at least low-degree remelting.

**Fig. 8:**
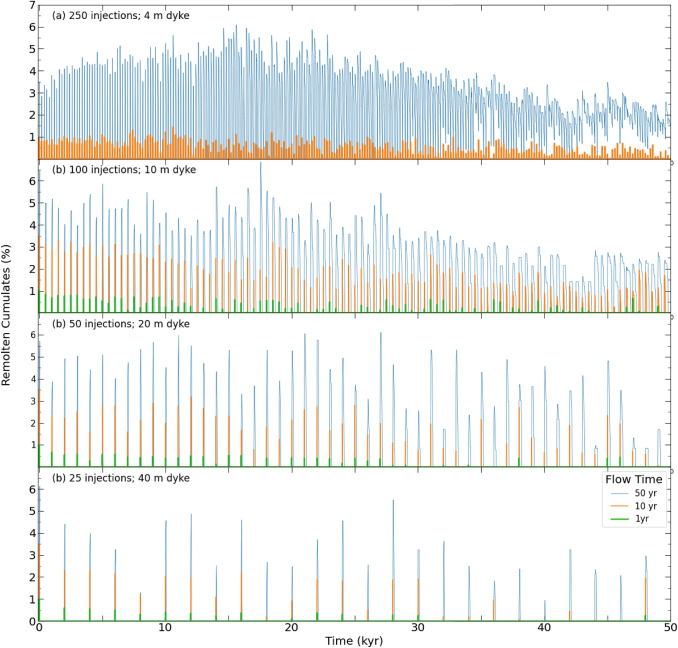
Evolution of the proportion of high-temperature cumulate melting (i.e., proportion of cumulates above 1050 °C) with time. Subplots are divided by injection width (as a function of injection frequency). Total volume is constant at 1000 m of injection

**Fig. 9:**
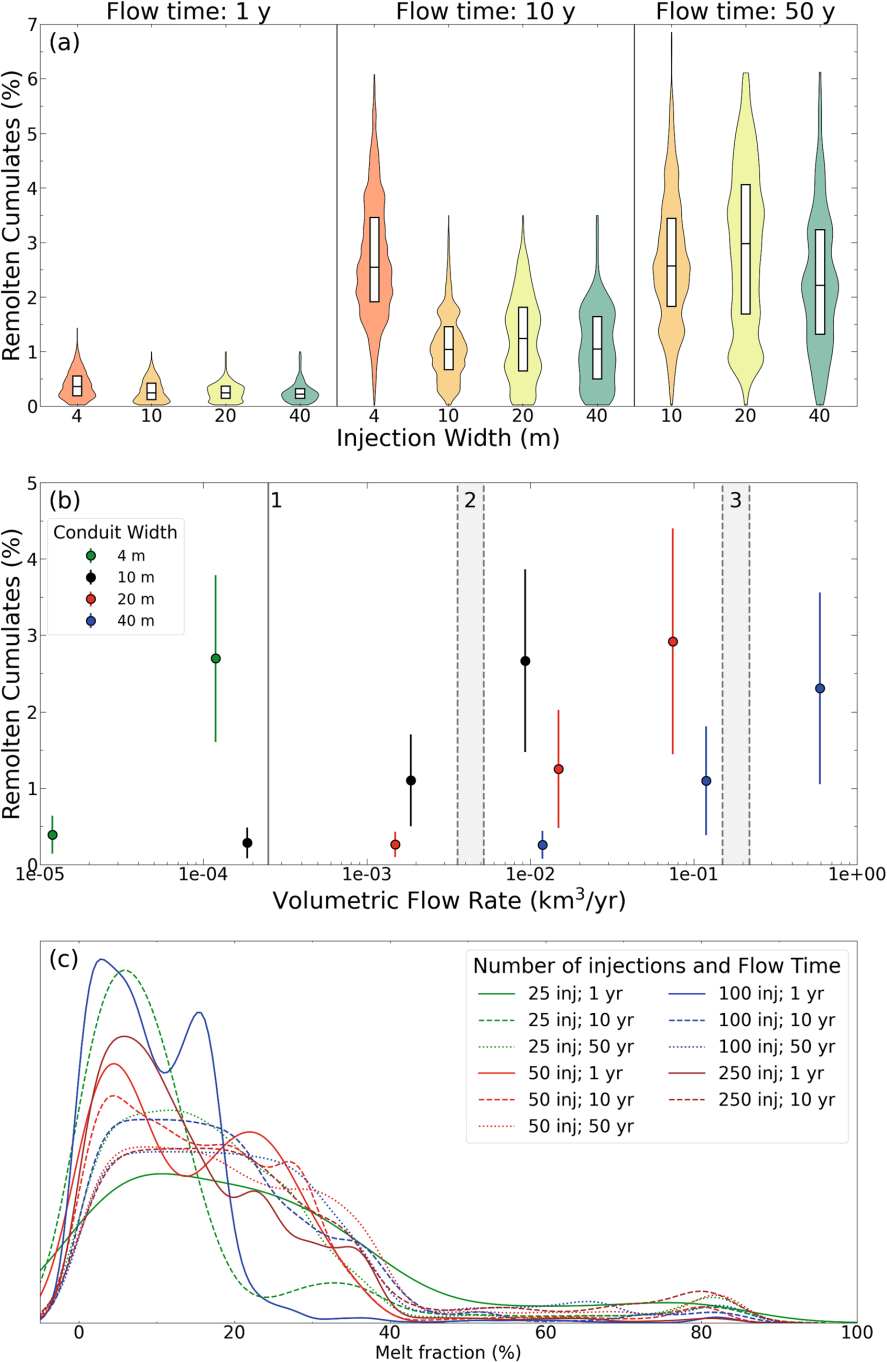
Results of thermal modelling of cumulate (re)melting. **a** Proportion of cumulates with temperature above 1050 °C in various simulations. Results are shown in a violin plot overlaid with a box indicating the median and interquartile range. **b** Proportion of cumulates with temperature above 1050 °C in different simulations vs. volumetric flow rate. Results are colour-coded for conduit width. Numbered grey bands indicate estimations of magmatic fluxes in: 1—Bear Valley intrusive suite (Klein et al. 2021); 2—average continental volcanoes (White et al. 2006); 3—Soufrière Hills volcano (Wadge et al. 2008); **c** Kernel density plot of melt fraction distribution in the cumulates for the last 10 kyr of each simulation

The temporal evolution of the proportion of high-T cumulate melts for different simulations is illustrated in Fig. 8. In all but one simulation, magma injection events result in an intermittent high-temperature cumulate melting event before returning to zero (i.e., temperatures below 1050 °C). This highlights the episodic nature of melting and the intimate association with the thermal input of each injection. The exception to this trend is the simulation with 250 injections (i.e., high intrusion frequency; see definition in Thermal Modelling Setup section) and a flow time of 10 years (intermediate flow time). Figure 9a summarises the results of Fig. 8 as a violin plot of the percentage of high-temperature cumulate melt produced through time in each simulation overlapped with the first, second (median), and third quartiles. The results indicate that the proportion of high-T cumulate melting increases with flow time (Fig. 9a). There are similar proportions of remolten cumulates for the different injection width scenarios at a given flow time, except for the high intrusion frequency simulation (i.e., salmon-coloured symbols) with a flow time of 10 years. This corresponds to the simulation where melts outlive repose periods between injections. We find, however, that the proportion of high-temperature cumulate melts never exceeds 7%, which can, therefore, be considered an upper limit. In higher dimensional models, given the higher surface-to-volume ratio favouring diffusive heat loss over heat addition by melt injection, even lower values can be expected.

The distribution of the lifetime of high-T cumulate melts (i.e., the time a part of the cumulate pile is above 1050 ºC) as a function of flow time and conduit thickness (inversely correlated with number of injections and injection frequency) is reported in Figure S8. The results show that higher flow times tend to result in a higher high-T cumulate melt lifetime; additionally, lifetime increases with injection frequency. A complete description of the results is available in the supplementary material. The results show that these high-temperature cumulate melts are transient in nature, never maintaining a temperature above 1050 °C for extended periods. The majority of the models brackets the lifetime between 1 and 100 years.

The proportion of high-T cumulate melting as a function of the flow rate and conduit width is illustrated in Fig. 9b. A given melt fraction of cumulate melting can be obtained by 4–5 orders of magnitude range in flow rate, which itself is dependent on the conduit width. In detail, a higher injection frequency of smaller injections leads to an increased melt fraction for a given flow time. We attribute these results to different factors. First, the increase in flow time for a given injection frequency leads to an increase in the flow to no-flow ratio, thereby increasing the time for heat accumulation. In addition, injections in this model are placed randomly in space, leading to a more efficient distribution of heat through the cumulate reservoir compared to a spatially fixed feeding system. Finally, the increase in cumulate melting with a higher number of smaller injections can be attributed to the increased ratio between surface area and volume, resulting in more efficient heat transfer. We emphasise that our model assumes conductive heat transfer exclusively, not allowing for mechanical interaction between basaltic–andesite injections and cumulates or advective heat transfer.

To investigate the relationship between the different parameters simulated (i.e., injection width and flow time) and the thermal stability, state and lifetime of the cumulate pile, we calculated the proportion of the cumulate body above solidus temperature (T > 850 °C) during the last 10 kyr of each simulation. Considering only the final part of the simulation allows quantification of the long-term effect of parameter variations well separated from initial conditions and initially transient model behaviour. The melt fraction distribution in the cumulates above the solidus temperature (modelled with the T–f relationship in Figure S2) is presented in Fig. 9c as a kernel density plot. A general outcome from all models is a significantly lower proportion of high melt fraction compared to low melt fraction, which translates into a predominance of low-T cumulate melting. Moreover, we note a sharp decrease in the abundance of supersolidus cumulates above ca. 40% melt fraction, corresponding to the change in the T–f slope (Figure S2). This implies that changes in the T–f slope, usually correlated with the appearance of new phases, may play a vital role in the melt fraction distribution in plutonic and subvolcanic settings, as suggested by Reubi and Blundy (2009).

A comparison of the proportion of supersolidus cumulates as a function of flow rate and frequency of intrusion (Figure S9) reveals a non-unique relationship. The proportion of supersolidus cumulates increases with the flow rate for a given intrusion frequency. Notably, the system exhibits a higher proportion of supersolidus cumulates with a higher intrusion frequency for a given flow time. The results are consistent with the observations of Spera and Bohrson (2018) and Calogero et al. (2020), who emphasised the often underrated role of intrusion frequency on the thermal evolution of the magmatic system and the potential for plutonic root recycling and assimilation.

## Discussion

### The nepheline-normative dykes are emplaced during the lifetime of the pluton

Most calculated amphibole–plagioclase equilibration temperatures range between 800 and 875 °C (Fig. 6). Experimentally determined phase equilibria indicate that the ne-normative dykes are either subsolidus or have very low melt fractions (below 5%) at these conditions (Fig. 7). The temperatures are similar to those calculated for the dioritic host rock by amphibole–plagioclase (Fig. 6; blue boxplot) and two-pyroxene thermometry (Ulmer et al. 1983). This indicates that the amphibole–plagioclase thermometer in the matrix of the ne-normative dykes record the temperatures in the dykes after complete equilibration of the latter with the dioritic host. Consequently, these temperatures correspond to the minimum host rock temperature during the intrusion of the dykes. Structural evidence and previous experimentally determined phase equilibria support this model: in a dioritic-tonalitic magma, residual melt fractions remain approximately constant between 50 and 35 wt.% upon cooling from 875 to 750 °C (Marxer and Ulmer 2019). Such melt fractions correspond to a rheological change in the dioritic–tonalitic host system, where it crosses the ductile–brittle transition (e.g., Vigneresse et al. 1996). The observation of brittle contacts between the ne-normative dykes and host rock and the backveining from the host dioritic mush (Fig. 2c) indicates a high strain rate upon emplacement, which occurs faster than the relaxation time (i.e., Maxwell time) of the host mush. Field observations of backveining are consistent with the emplacement of the ne-normative melts as dykes during the suprasolidus lifetime of the pluton.

### The nepheline-normative dykes cannot be products of crystal fractionation

**Fig. 10:**
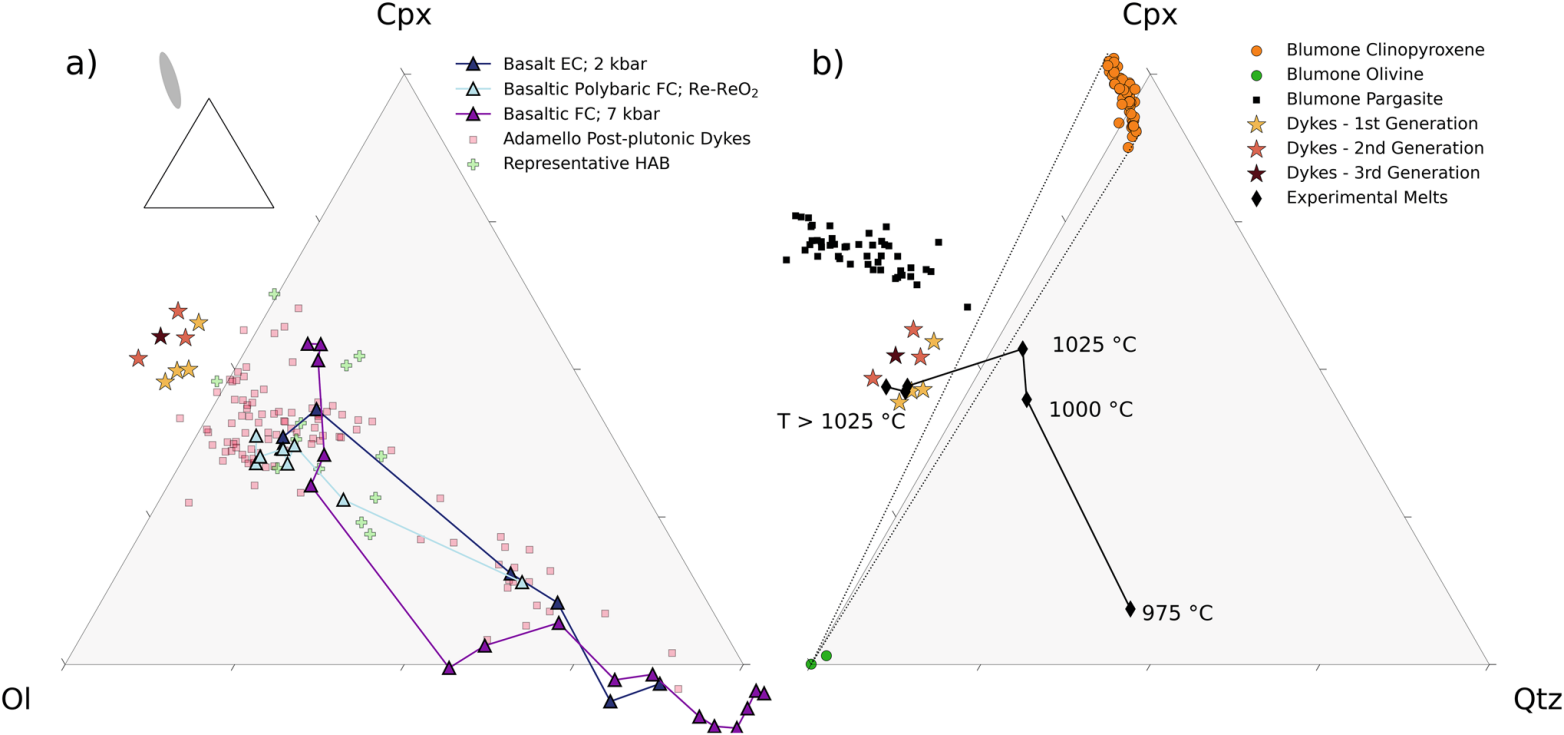
Olivine–clinopyroxene–quartz pseudo-ternary diagrams, projected from plagioclase, K-feldspar, apatite, and Fe–Ti oxide, after Grove (1993). **a** Ne-normative dyke compositions plotted together with experimental and natural rock compositions (see caption of Fig. 3 for references). The small inset shows the normative composition of the ultra-calcic, nepheline-normative melts (Médard et al. 2006) relative to the ol-cpx-qtz diagram. **b** Experimental liquids of this study, supplemented by natural clinopyroxene, olivine, and amphibole compositions from the Blumone complex and the ne-normative dykes. The two dotted lines represent the maximum spread of the olivine–clinopyroxene tie-line considering the compositional range of these phases. The following abbreviations were used: *EC* equilibrium crystallisation, *FC* fractional crystallisation, *HAB* high-alumina basalt

Despite their low SiO_2_ contents (< 47 wt.%), the ne-normative dykes do not represent primitive calc-alkaline magmas. This is evident from the low MgO (4.9–7.0 wt.%) and Ni (13–41 μg/g) contents when compared to mantle-derived melts (e.g., Schmidt and Jagoutz 2017). The olivine–clinopyroxene–quartz pseudo-ternary diagram (Fig. 10a), projected from plagioclase (after Grove 1993), reveals that the Blumone ne-normative dykes have negative quartz component. This is at odds with the positive quartz components of primary calc-alkaline magmas and experimentally determined liquid lines of descent (Nandedkar et al. 2014; Marxer et al. 2022, 2023). In addition, we plot compositions of post-plutonic dykes of the Southern Adamello, which correspond to the liquid line of descent of the Re di Castello super-unit (and of calc-alkaline differentiation; Hürlimann et al. 2016), as well as representative compositions of high-alumina basalts (Sisson and Grove 1993b), derived from mid- to lower crustal fractional crystallisation of primitive hydrous magmas (Sisson and Grove 1993a,b; Pichavant and Macdonald 2007). Both groups broadly overlap with the experimental liquid lines of descent of basaltic arc magmas (Fig. 10a).

The present phase equilibria experiments indicate that plagioclase and hercynitic spinel are near-liquidus phases of the ne-normative melts at 1050–1075 °C (Fig. 7). This finding contrasts with low-pressure, mafic calc-alkaline experimental studies, where olivine ± Cr-bearing spinel are the first phases to saturate (e.g., Sisson and Grove 1993a; Blatter et al. 2013; Marxer et al. 2023).

Finally, trace-element contents of the ne-normative dykes do not agree with a fractionation hypothesis. There are significant differences compared with the post-plutonic Adamello basaltic dykes. For example, the superchondritic Nb/Ta of the ne-normative dykes contrasts with the lower ratio for the post-plutonic dykes (Fig. 4b). In addition, the LILE depletion (Fig. 4b) and convexity (i.e., λ_2_ < 0 after O’Neill 2016) of the chondrite-normalised REE pattern (Fig. 4a) of the ne-normative dykes are unexpected along a calc-alkaline LLD (e.g., Hürlimann et al. 2016). In conclusion, the major- and trace-element chemistry of the ne-normative dykes is inconsistent with a calc-alkaline differentiation trend, and thus, they are not the product of crystallisation differentiation processes of primitive arc magmas.

### The nepheline-normative dykes as products of gabbroic cumulate melting

Based on field relations and the similarity of plagioclase between the Blumone cumulates and the ne-normative dyke phenocrysts, we evaluate the hypothesis that ne-normative dykes are the products of amphibole-gabbro melting processes.

Melting of an amphibole gabbroic cumulate is consistent with the SiO_2_-undersaturated nature of the ne-normative dykes, considering the common presence of pargasite–tschermakite. Given the peritectic nature of amphibole breakdown in mafic systems (e.g., melt + cpx + ol + sp = amphibole; Blatter et al. 2017), the petrogenesis of the ne-normative dykes implies not only a thermal perturbation outside the amphibole stability field but also outside the stability of olivine and clinopyroxene. This is illustrated by chemographic relationships (Fig. 10b), where the formation of nepheline-normative compositions implies crossing the olivine–clinopyroxene tie-line. The destabilisation of the Fe–Mg silicates is, thus, critical in generating ne-normative melts.

To quantify the stoichiometry of the melting reaction, we performed boot-strapped Monte Carlo simulations on the mass balance between the ne-normative dykes and the mineralogy of the Blumone cumulates. We modelled a melt-amphibole–plagioclase–spinel reaction simulating a temperature increase in the order of 100 °C, starting from 950 to 975 °C. The calculated stoichiometry is (factors with 1 s.d.)

1 $$0.62\left( 2 \right)amph + 0.39\left( 2 \right)plag = 1 melt + 0.01\left( 1 \right) spinel$$

In reaction (1), the melting of an amphibole (pargasite–tschermakite) and plagioclase (An_90_) assemblage results in nepheline-normative melt with a small amount of hercynitic spinel. This is consistent with our experimentally determined phase equilibria, where SiO_2_-undersaturated melt is in equilibrium with spinel and plagioclase at the liquidus but not saturated in Fe–Mg silicates.

The bulk-rock geochemistry further supports the genesis of the ne-normative dykes from amphibole-gabbro melting. The elevated Na_2_O contents of these dykes (1.7–3.0 wt.%; Fig. 3d and Table 1) and the silica-undersaturated chemistry (Fig. 10) point to an amphibole control. This is further supported by the elevated ratios of Nb/Ta in the ne-normative dykes. Nb is preferentially incorporated over Ta in high Mg# amphibole (Nandedkar et al. 2016). Elevated Nb/Ta ratios are recorded by Blumone pargasites (see supplementary material), consistent with the hypothesis that melting of Blumone amphibole will result in elevated Nb/Ta of ne-normative dykes. The hump-shaped middle REE content of the nepheline-normative dykes compared to common calc-alkaline differentiates is additional evidence of amphibole melting (Nandedkar et al. 2016). The involvement of plagioclase is reflected by the high Al_2_O_3_ and CaO contents (Fig. 3) and the weak positive Eu anomalies (Fig. 4a). Finally, the negative anomalies in P, Zr, Hf, Th, and the depletion in LILE compared to the post-plutonic basaltic dykes (Hürlimann et al. 2016) are consistent with a cumulate signature of the dyke source.

### Timescales of cumulate melting

The extraction timescales of the cumulate melting products (i.e., ne-normative melts) and segregation until emplacement in the studied dyke suite remain uncertain due to the absence of direct constraints. However, our thermal model, tailored to this case study, provides constraints on the maximum in situ thermal lifetime of cumulate melting products. Most of our simulations show that cumulate melting products can thermally persist on the order of decades (Figure S8). These decadal timescales of cumulate melting align within the same order of magnitude as the diffusion study of Costa and Dungan (2005). In their study, the authors show that the thermochemical barriers to phlogopite–hornblende gabbro cumulate melting in the Tatara-San Pedro complex are low, with fast xenolith digestion. This is especially interesting considering the high enthalpic cost of peritectic melting reactions (e.g., Blatter et al. 2017), attesting to the high thermal throughput in the parts of the reservoirs where cumulate melting occurs. Such a situation is comparable to the Blumone complex case study, since amphibole plays a critical role in the petrogenesis of these ne-normative dykes. Moreover, the recognition of cumulate melting processes in volcanic deposits (e.g., Schiano et al. 2000; Dungan and Davidson 2004; Reubi et al. 2011) suggests that cumulate melting and digestion occur at timescales substantially faster than the lifetime of magmatic systems.

Field observations indicate rapid emplacement of the ne-normative dykes, evident from the sharp contacts with the partially molten host rock (Fig. 2). Assuming characteristic lengths of ne-normative melt extraction pathways from the cumulate pile from 400 to 1000 m and maximum thermal lifetimes of 1 to 100 years constrained by our thermal modelling, melt segregation rates during extraction, and emplacement can be constrained to no less than 1.6 × 10^–7^ to 3.2 × 10^–5^ m/s (4–1000 m/yr). These values are consistent with the established range of melt segregation rates by channelised flow through partially molten systems (Katz et al. 2022) followed by brittle fracture-bound emplacement in the surrounding diorite. The present study does not provide sufficient constraints to further investigate the challenging fluid mechanics problem of such relatively rapid extraction of intermittently formed cumulate melts.

### Cumulate melting in arc volcanoes

The experimentally determined phase equilibria for the ne-normative dykes imply that they were generated at temperatures of at least 1050 °C (under H_2_O-saturated conditions). Such high temperatures in an upper crustal setting are restricted to an active magmatic plumbing system with sufficient heat supply (e.g., Tornare et al. 2018). This is consistent with the previous interpretations of the Blumone complex as a subvolcanic magmatic system and testified by extraordinarily high contact metamorphic temperatures exceeding 850 °C (Ulmer 1982).

The range of evidence from nearly undetectable (e.g., Llaima; Reubi et al. 2011) to highly evident (e.g., Blumone, this study) suggests that mafic root recycling is a common, albeit at times cryptic, process in transcrustal magmatic arc systems where heat and mass transfers modulate crust construction, magmatic reservoir assembly, and volcanic activity. In addition, while our study demonstrates that high-temperature cumulate melting does not represent a volumetrically significant differentiation process, it remains essential to consider its significance in shaping the chemical diversity of arc magmas.

Moreover, the identification of such processes in the field sheds new light on understanding the magmatic record of systems where their geometry or the representativity of melt compositions is unclear, such as melt inclusions, where such compositions can be preserved prior to mixing processes or reaction with other physical components within a magmatic system.

## Conclusions

**Fig. 11:**
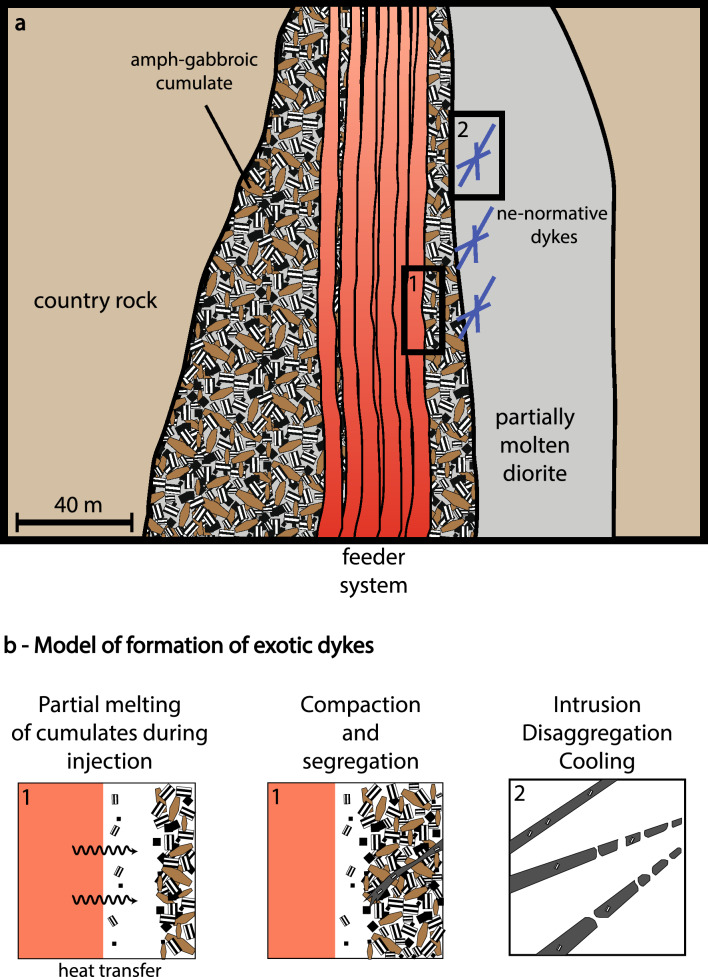
Schematic sketch illustrating the proposed model for the Blumone ne-normative dyke generation. **a** Schematic drawing of the Blumone feeder system position relative to the adjacent diorites and the location of the ne-normative dykes, as observed in the field. Rectangles correspond to the sketches drawn below. **b** The generation of the ne-normative dykes starts with the partial melting of amphibole gabbroic cumulates by heat transfer from the feeder system during magma injection. This is followed by compaction and segregation of the ne-normative melts from the amphibole gabbroic source, partially entraining cumulate cargo (i.e., “phenocrysts”), and intrusion into the adjacent partially molten diorites, where they disaggregate and rapidly cool to the ambient temperature of the diorites

The Southern Adamello Blumone complex is a key locality for understanding the formation of ne-normative magmas in volcanic plumbing systems (Fig. 11). The detailed petrological study of ne-normative dykes reveals the mechanical entrainment of cumulate crystals into alkaline melts. Saturation experiments on the exposed ne-normative dykes indicate that these are most likely a melting product of amphibole-gabbro cumulates. Therefore, ne-normative arc magmas could represent the product of cumulate remelting and not a product of crystallisation–differentiation of unidentified alkaline magmas. The availability of amphibole- or phlogopite-bearing cumulates in subvolcanic feeder zones is a key feature for remelting processes, favouring assimilation, disaggregation, and partial melting. Experimental data combined with thermal modelling indicate that high-T cumulate melting does not produce voluminous products but can contribute significantly to the diversity of arc melt compositions. Even though high-T cumulate melting is unlikely to be a volumetrically major process operating in subvolcanic mafic roots, the findings of ne-normative magmas in subvolcanic plumbing systems provide a fingerprint for prevailing high temperatures with potentially multiple replenishment episodes. The results further predict that low-T cumulate remelting may be widespread in transcrustal magmatic systems containing hydrous phases. Future studies on melt inclusions in volcanic deposits, where the geometry of the subvolcanic roots cannot be investigated in the same detail, are a promising avenue to further investigate the mechanism of high-T cumulate melting.

### Supplementary Information

Below is the link to the electronic supplementary material.

Supplementary file1 (XLSX 166 KB)

Supplementary file2 (XLSX 103 KB)

Supplementary file3 (XLSX 37 KB)

Supplementary file4 (XLSX 23 KB)

Supplementary file5 (DOCX 2818 KB)

## Data Availability

Data established in the scope of this project are available as electronic supplementary material to this manuscript.
