# Supplementary material for: Alkaline magmas in shallow arc plutonic roots: a field and experimental investigation of hydrous cumulate melting in the southern Adamello batholith
Source: Contrib Mineral Petrol. 2023 Sep 3;178(9):64. doi: 10.1007/s00410-023-02047-3 (PMC11008074; doi:10.1007/s00410-023-02047-3)
Supplement: Supplementary file 5 — Supplementary file5 (DOCX 2818 KB) [file 410_2023_2047_MOESM5_ESM.docx]

**Electronic Supplementary Material**

**Alkaline magmas in shallow arc plutonic roots: A field and experimental investigation of hydrous cumulate melting in the Southern Adamello Batholith**

Manuel Pimenta Silva^1,*^ ⋅ Felix Marxer^1,2^ ⋅ Tobias Keller^1^ ⋅ Andrea Giuliani^1^ ⋅ Peter Ulmer^1^ ⋅ Othmar Müntener^3^

^1^ Institute of Geochemistry and Petrology, ETH Zürich, Clausiustrasse 25, 8092 Zürich, Switzerland

^2^ Institute of Mineralogy, Leibniz University Hannover, Callinstraße 3, 30167 Hannover, Germany

^3^ Institute of Earth Sciences, Université de Lausanne, Switzerland

*Corresponding author: [manuel.dossantos@erdw.ethz.ch](mailto:manuel.dossantos@erdw.ethz.ch)

This Electronic Supplementary Material contains complementary information on the mineral chemistry of natural and experimental samples, as well as supplemental figures.

# A remark on the experimental approach

Despite the hypothesis being tested in this contribution, we chose not to utilise the Blumone amphibole gabbro as a starting material given the pervasive subsolidus alteration of these samples, with high-Si amphibole, sericite and epidote. These phases, resulting from pneumatolytic activity, increase the fusibility of the system, but do not represent the composition of the cumulate during the lifetime of the magmatic system. As such, only a saturation approach was performed to constrain near-liquidus phase relations.

# Calculation of volumetric flow rates

Volumetric flow rates were calculated based on the equation for Poiseuille flow by Delaney & Pollard (1982) for circular conduits:

$$Q= \frac{\pi}{8}\frac{\Delta P}{\eta L}R^{4}$$

Where Q corresponds to the volumetric flow rate (m^3^/s), $\Delta P/L$ corresponds to the driving pressure gradient (MPa/m), $\eta$ corresponds to the viscosity (Pa s), and R corresponds to the radius of the conduit (m). We assume a $\Delta P$ = 12 MPa (assuming $\Delta\rho$ = 300 kg/cm^3^), viscosity of 100 Pa s. The radius corresponds to the half-width of the conduits.

# Mineral Chemistry

## Cumulate vs phenocrysts

### Clinopyroxene

Clinopyroxene mineral chemistry was recalculated into cation units (atoms per formula unit, a.p.f.u.) based on the method of Wood & Banno (1973). Cumulate clinopyroxene is a low-Al_2_O_3_ (<3.5 wt.%) diopside (Figure S1), as previously observed by Ulmer et al. (1983). Phenocrystic clinopyroxene compositions overlap with the cumulate counterparts. In one sampled dyke (B17-8), the phenocrystic clinopyroxene has lower Mg# than in other dykes and most cumulate clinopyroxenes. The same sample also tends to have the lowest Al^IV^ of the measured crystals.

This distinction into several groups of clinopyroxene phenocrysts is also observed in their REE pattern (Figure S3). The three groups display MREE enrichment relative to the light and heavy REE. The first clinopyroxene phenocryst group REE pattern (MP18-8 and MP18-14) overlaps with that of cumulate clinopyroxene. In contrast, the B-17-8 clinopyroxene shows a more evolved REE pattern (i.e., enriched) with a more pronounced Eu anomaly, consistent with its derivation from the intruded Blumone quartz-diorites.

### Amphibole

Similar to plagioclase and clinopyroxene, amphibole phenocrysts and the ones in the amphibole gabbroic cumulates share common features. Both groups display decreasing edenite component with increasing Si (c.p.f.u.), as observed in Figure S4a. This trend is expected in calc-alkaline systems, where plagioclase is co-crystallising; in other words, with increasing differentiation (i.e., Si in amphibole) and lower temperature, amphiboles show progressively lower A-site occupancy (Blundy & Holland, 1990).

The REE profiles of amphibole were divided according to the amphibole classification scheme (Figure S2b-d). Cumulate pargasite shows a wide range of (Sm/La)_N_. The decrease of (Sm/La)_N_ coincides with Si (c.p.f.u.) increase. This is related to the D_Sm_/D_La_ increase with decreasing temperature and increasing Si (c.p.f.u.) (Nandedkar et al., 2016). The phenocrystic amphibole REE concentrations overlap with those of the cumulate. However, similar to clinopyroxenes, the most evolved amphibole crystals observed, the Mg-hornblende phenocrysts of sample B-17-8, are more enriched in REE, with a more pronounced negative Eu anomaly. This again, is suggestive for its derivation from the Blumone quartz-diorites.

## Matrix

The matrix amphiboles are dominantly tschermakitic (Figure S5a), with a lower edenite component range than the Blumone cumulates (Figure S2). The anorthite content of the matrix plagioclases of the ne-normative dykes are plotted in Figure S3b. Most of the plagioclase is bytownitic, apart from samples MP18-15 and B17-8 that are somewhat lower. There is petrographic evidence for the co-crystallisation of amphibole and plagioclase by inclusion systematics.

# Experiments

## Phase compositions

Residual **melt** (Figure S4) was always present in sufficiently high fractions (> 33 wt.%) to enable EPMA measurements with a 20 μm beam diameter. This minimised alkali diffusion during analysis as later confirmed by mass balance calculations.

The decreased in Al_2_O_3_, SiO_2_ and CaO, and increase of FeO and MgO (Figure S7) of the experimental melts at near-liquidus conditions are dictated by Al-rich spinel and An-rich plagioclase saturation. Residual liquids at 1075 and 1050 °C display similar compositions, owing to the minimal change in phase proportions and composition.

The saturation of olivine and increased plagioclase and spinel proportions lead to a considerable increase in SiO_2_ content (ca. 4 wt.%) and decreased MgO and FeO. This significant FeO change is attributed to the distinct increase of the magnetite component in the spinel phase changing from hercynitic spinel to aluminous titano-magnetite. The increasing SiO_2_ trend continues at 1000 °C. The saturation of clinopyroxene dictates the decrease in CaO content, which is also evident for MgO and FeO.

The saturation of amphibole at 975 °C further increases the melt SiO_2_ content, as predicted by phase equilibria in calc-alkaline systems (e.g., Yoder & Tilley, 1962; Green & Ringwood, 1968; Cawthorn & O’Hara, 1976).

K_2_O and P_2_O_5_ melt concentrations increase with progressive differentiation as a function of residual melt fraction, owing to their incompatible behaviour in this temperature range. Na_2_O displays a similar trend with decreasing temperature since it is incompatible with crystallising phases (crystallising plagioclase is very anorthite-rich).

The anorthite content of the experimental **plagioclases** is listed in Table 2. The anorthite content of the plagioclase exceeds 0.9 from 1000 to 1075 °C, decreasing to 0.88 at 975 °C. The high anorthite content in the reported experiments is a consequence of three factors: High aH_2_O results in the destabilisation of the albite melt component and reduction of the silica activity, favouring high anorthite plagioclase (Johannes, 1978; Arculus & Wills, 1980). The decreasing SiO_2_ activity at low pressure (e.g., Carmichael, 2004) also favours higher anorthite contents, by decreasing the CaTs activity in the melt in favour of the An activity (e.g., Grove et al., 2003). In addition, the high Al_2_O_3_/SiO_2_ and CaO/Na_2_O of the nepheline-normative dykes promotes the crystallisation of anorthitic plagioclase. Such compositional effects are confirmed when comparing the higher K_D_ _plag-melt_ ^Ca-Na^ of a high-alumina basalt (Baker & Eggler, 1987; Sisson & Grove, 1993a; Panjasawatwong et al., 1995) with the lower values in a low-alkali tholeiite (Takagi et al., 2005).

**Spinel** is stable in all subliquidus experimental runs. At high temperatures (1050 and 1075 °C), the spinel in equilibrium with silicate melt and plagioclase is enriched in MgO and Al_2_O_3_ compared to FeO and Fe_2_O_3_ (hercynitic, Table 3). The temperature decrease and onset of olivine crystallisation at 1025 °C explain the stabilisation of the magnetite component and decrease of Mg-bearing endmembers since MgO is partitioned into olivine. In addition, the Kd(Fe-Mg) olivine-spinel decreases with decreasing temperature and Al content of the spinel. The xFe increase throughout the studied temperature range also illustrates the higher compatibility of MgO over FeO in silicate phases.

**Olivine** crystallises at and below 1025 °C. At 1025 and 1000 °C, the xMg is approximately constant, around 0.82. At 975 °C, there is a considerable decrease to 0.67. The lower temperature, progressive crystallisation of Fe-Mg silicate phases with a higher affinity for MgO, and the destabilisation of magnetite upon amphibole crystallisation contribute to the change in olivine chemistry.

**Clinopyroxene** is stable at 1000 and 975 °C. The clinopyroxene has a diopsidic to augitic composition. At 1000 °C, the clinopyroxene is sector-zoned and the low-Al sector has 4.3±0.3 wt.% Al_2_O_3_ and a dn xMg of 0.80±0.01, while the high-Al sector has 8.3±0.8 wt.% Al_2_O_3_ and an xMg of 0.73±0.02. The experimental clinopyroxene at 975 °C is homogeneous, with 6.1±0.4 wt.% Al_2_O_3_ and xMg of 0.67±0.02.

**Amphibole** is observed at 975 °C and has a pargasitic composition (Si = 6.03±0.04 a.p.f.u.; (Na+K)^A^=0.68±0.03 a.p.f.u.).

Supplementary Figure 1 - Geometry of the thermal model.

Supplementary Figure 2 - Melt fraction variation with temperature from the experiments of Marxer et al. (2023) on a high-Al basalt at 200 MPa and fitting function used for thermal modelling simulations.


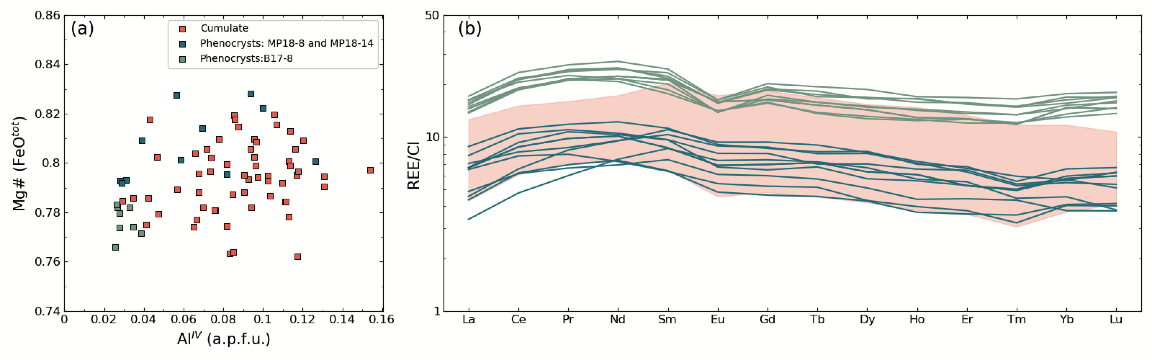


Supplementary Figure 3 - Comparison of the composition of phenocrystic (blue, green) and cumulate (red) clinopyroxene. (a) Mg#-Al^IV^ systematics. (b) Chondrite-normalised (McDonough & Sun, 1995) REE contents. Red band corresponds to range of cumulate clinopyroxene contents.


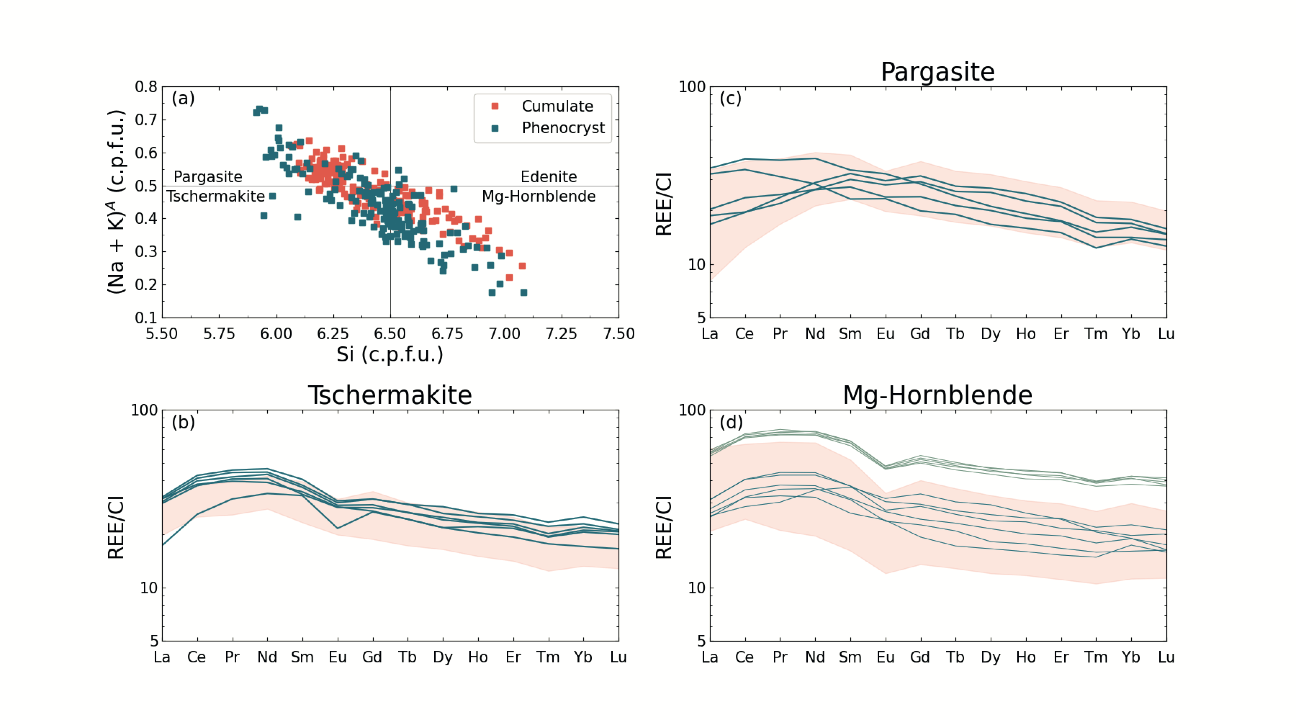


Supplementary Figure 4 - Comparison of major element compositions of phenocrystic (blue) and cumulate (red) amphibole. (a) Silica vs edenite component. Chondrite-normalised (McDonough & Sun, 1995) REE content of (b) tschermakite, (c) pargasite and (d) Mg-hornblende. The red bands in subplots b,c,d correspond to the measured content range in cumulate amphibole.

Supplementary Figure 5 - Composition of matrix amphibole and plagioclase in nepheline-normative dykes. (a) Classification of amphibole according to silica content and A-site occupancy. Samples are colour-coded according to the boxplot colours in (b). Amphibole composition is calculated considering charge balance (no assumption on Fe valence state) and Na^M4^ = 0. (b) Boxplot of anorthite content of matrix plagioclase.


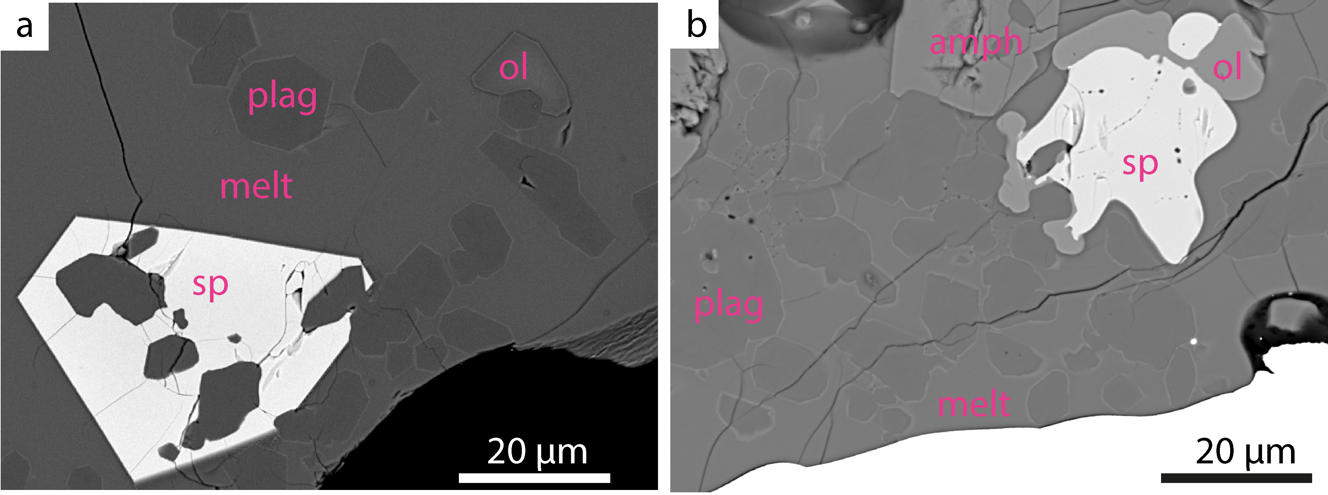


Supplementary Figure 6 - BSE images of experimental run products. a) Experimental run at 1025 ºC, showing melt in equilibrium with unzoned plagioclase (plag), spinel (sp) and zoned olivine (ol). b) Experiment at 975 ºC, showing melt in equilibrium with unzoned plagioclase, spinel, olivine and amphibole (amph).

Supplementary Figure 7 - Compositional variation of experimental melts (wt. % oxides) as a function of temperature.


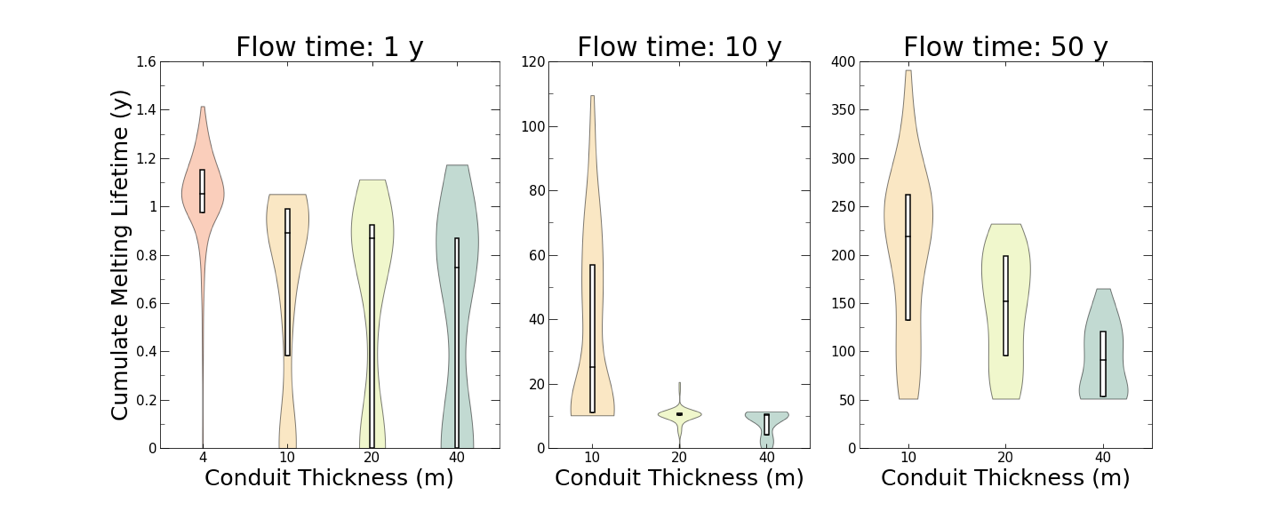


Supplementary Figure 8 – Cumulate melting lifetime (defined as the time range when the cumulate complex is above 1050 ºC, defined as the minimum temperature to generate ne-normative melts, based on the experimental results) distribution in various simulations. Subplots show simulations of different flow time. Results are shown in a violin plot overlaid with a box indicating the median and interquartile range. There is a direct relationship between the lifetime of these melts and the flow time, whereby higher flow times tend to result in a higher high-T cumulate melt lifetime. Secondly, there is an increase in the lifetime with lower injection thickness due to the inverse relationship between injection thickness and frequency, such that lower injection thickness increases the flow to no-flow ratio. At low intrusion frequency (i.e., larger conduits) and/or low flow time, we observe that the lifetime of high-T cumulate melts is lower than the flow time. This observation implies that the heat loss during no-flow periods is more significant than the heat gained during flow periods.

Supplementary Figure 9 - Variation of proportion of cumulates above solidus temperature vs. volumetric flow rate in the last 10 ky. Simulations are colour-coded by injection width. Arrows intend to show the effect of flow time (for constant width) and intrusion frequency (for constant flow time).

# References

Arculus, R. J., & Wills, K. J. A. (1980). The petrology of plutonic blocks and inclusions from the lesser antilles Island arc. *Journal of Petrology*, *21*(4), 743–799. https://doi.org/10.1093/petrology/21.4.743

Baker, D. R., & Eggler, D. H. (1987). Compositions of anhydrous and hydrous melts coexisting with plagioclase, augite, and olivine or low-Ca pyroxene from 1 atm to 8 kbar: application to the Aleutian volcanic center of Atka. *American Mineralogist*, *72*(1–2), 12–28.

Carmichael, I. S. E. (2004). The activity of silica, water, and the equilibration of intermediate and silicic magmas. *American Mineralogist*, *89*(10), 1438–1446. https://doi.org/10.2138/am-2004-1011

Cawthorn, R. G., & O’Hara, M. J. (1976). Amphibole fractionation in calc-alkaline magma genesis. *American Journal of Science*, *276*(3), 309–329. https://doi.org/10.2475/ajs.276.3.309

Green, T. H., & Ringwood, A. E. (1968). Genesis of the calc-alkaline igneous rock suite. *Contributions to Mineralogy and Petrology*, *18*(2), 105–162. https://doi.org/10.1007/BF00371806

Grove, T. L., Elkins-Tanton, L. T., Parman, S. W., Chatterjee, N., Müntener, O., & Gaetani, G. A. (2003). Fractional crystallization and mantle-melting controls on calc-alkaline differentiation trends. *Contributions to Mineralogy and Petrology*, *145*(5), 515–533. https://doi.org/10.1007/s00410-003-0448-z

Johannes, W. (1978). Melting of Plagioclase in the System Ab-An-H20 at PH2O=5 kbars , an Equilibrium Problem. *Contributions to Mineralogy and Petrology*, *303*, 295–303.

Marxer, F., Ulmer, P., & Müntener, O. (2023). Ascent-driven differentiation: a mechanism to keep arc magmas metaluminous? *Contributions to Mineralogy and Petrology*, *178*(8), 51. https://doi.org/10.1007/s00410-023-02035-7

Nandedkar, R. H., Hürlimann, N., Ulmer, P., & Müntener, O. (2016). Amphibole–melt trace element partitioning of fractionating calc-alkaline magmas in the lower crust: an experimental study. *Contributions to Mineralogy and Petrology*, *171*(8–9), 1–25. https://doi.org/10.1007/s00410-016-1278-0

Panjasawatwong, Y., Danyushevsky, L. V., Crawford, A. J., & Harris, K. L. (1995). An Experimental-Study of the Effects of Melt Composition on Plagioclase - Melt Equilibria at 5-Kbar and 10-Kbar - Implications for the Origin of Magmatic High-an Plagioclase. *Contributions to Mineralogy and Petrology*, *118*(4), 420–432. https://doi.org/DOI 10.1007/s004100050024

Sisson, T. W., & Grove, T. L. (1993). Experimental investigations of the role of H2O in calc-alkaline differentiation and subduction zone magmatism. *Contributions to Mineralogy and Petrology*, *113*(2), 143–166. https://doi.org/10.1007/BF00283225

Takagi, D., Sato, H., & Nakagawa, M. (2005). Experimental study of a low-alkali tholeiite at 1-5 kbar: Optimal condition for the crystallization of high-An plagioclase in hydrous arc tholeiite. *Contributions to Mineralogy and Petrology*, *149*(5), 527–540. https://doi.org/10.1007/s00410-005-0666-7

Ulmer, P., Callegari, E., & Sonderegger, U. C. (1983). Genesis of the mafic and ultramafic rocks and their genetical relations to the tonalitic-trondhjemitic granitoids of the southern part of the Adamello Batholith, (Northern Italy). *Mem Soc Geol Ital*, *26*(November), 171–222. http://scholar.google.com/scholar?hl=en&btnG=Search&q=intitle:Genesis+of+the+mafic+and+ultramafic+rocks+and+their+genetical+relations+to+the+tonalitic-trondhjemitic+granitoids+of+the+southern+part+of+the+Adamello+Batholith,+(Northern+Italy)#0

Wood, B. J., & Banno, S. (1973). Garnet-Orthopyroxene and Orthopyroxene-Clinopyroxene Relationships in Simple and Complex Systems. *Contributions to Mineralogy and Petrology*, *24*, 109–124.

Yoder, H. S., & Tilley, C. E. (1962). Origin of basalt magmas: An experimental study of natural and synthetic rock systems. *Journal of Petrology*, *3*(3), 342–532. https://doi.org/10.1093/petrology/3.3.342
